# Supplementary material for: Associations of social environment, socioeconomic position and social mobility with immune response in young adults: the Jerusalem Perinatal Family Follow-Up Study
Source: BMJ Open. 2017 Dec 21;7(12):e016949. doi: 10.1136/bmjopen-2017-016949 (PMC5778288; doi:10.1136/bmjopen-2017-016949)
Supplement: Supplementary file 1 [file bmjopen-2017-016949supp001.pdf]

Supplement 1. Diamedix Immunosimplicity Is-CMV IgG Test Kit

(Diamedex Corporation, 2008)

The Test Kit is intended for the qualitative and semi-quantitative detection of antibodies to CMV antigen. Compared to currently used testing methods in three different United States-based laboratories, the sensitivity and specificity of the Diamedix Kit ranged from 99.3%-100.0% and 87.7%-100.0% respectively, and overall agreement ranged from 97.0%-99.0%. Seroprevalence results detected using this kit were in agreement with prevalence rates reported in United States blood donor populations.

Diamedix. CMV IgG: Enzyme Immunoassay Test Kit. 2008;3–6.
