# Supplementary material for: Associations of social environment, socioeconomic position and social mobility with immune response in young adults: the Jerusalem Perinatal Family Follow-Up Study
Source: BMJ Open. 2017 Dec 21;7(12):e016949. doi: 10.1136/bmjopen-2017-016949 (PMC5778288; doi:10.1136/bmjopen-2017-016949)
Supplement: Supplementary file 2 [file bmjopen-2017-016949supp002.pdf]

Supplement 2. Correlations between SEP-related variables in the JPS Family Follow-up cohort

	Father's occupation[a]	Maternal education[b]	Maternal religiosity[c]	Paternal lay-leadership[c]	Number of siblings[b]	Offspring occupation[a]	Offspring education[b]	Offspring religiosity[c]	Offspring parity[b]
Father's occupation[a]	1.00								
Maternal education[b]	0.52	1.00							
Maternal religiosity[c]	0.23	0.07	1.00						
Paternal lay-leadership[c]	0.32	0.10	0.49	1.00					
Number of siblings[b]	0.10	-0.14	0.54	0.60	1.00				
Offspring occupation[a]	0.22	0.29	0.00	0.02	-0.10	1.00			
Offspring education[b]	0.30	0.32	0.19	0.22	0.08	0.38	1.00		
Offspring religiosity[c]	0.19	0.06	0.72	0.47	0.53	0.00	0.18	1.00	
Offspring parity[b]	0.14	-0.04	0.50	0.49	0.54	-0.05	0.06	0.57	1.00

[a]In 6 categories; Spearman's rank correlation coefficient
[b]Continuous; Pearson correlation coefficient
[c]Dichotomous; Pearson correlation coefficient
