# Supplementary material for: Associations of social environment, socioeconomic position and social mobility with immune response in young adults: the Jerusalem Perinatal Family Follow-Up Study
Source: BMJ Open. 2017 Dec 21;7(12):e016949. doi: 10.1136/bmjopen-2017-016949 (PMC5778288; doi:10.1136/bmjopen-2017-016949)
Supplement: Supplementary file 3 [file bmjopen-2017-016949supp003.pdf]

Supplement 3. Principal Component Analysis Loadings

Childhood			Adulthood		
	Component Loadings			Component Loadings	
Variables Included	Household	Socioeconomic	Variables Included	Household	Socioeconomic
Paternal Occupation	0.112	0.656	Offspring Occupation	-0.111	0.719
Maternal Education	-0.132	0.723	Offspring Education	0.115	0.690
Maternal Religiosity	0.538	0.048	Offspring Religiosity	0.695	0.056
Paternal Lay Leadership	0.550	0.119			
Number of Siblings	0.616	-0.180	Offspring Parity	0.701	-0.065
Variance Explained	74%		Variance Explained	75%	

Principal Component Score Distribution

	Mean	SD	Minimum	Maximum
**Household Components**				
Childhood	-0.003	1.49	-4.63	1.86
Adulthood	-0.008	1.27	-4.05	2.08
**Socioeconomic Components**				
Childhood	0.04	1.21	-4.14	3.52
Adulthood	0.06	1.15	-3.66	3.02
