# Supplementary material for: Associations of social environment, socioeconomic position and social mobility with immune response in young adults: the Jerusalem Perinatal Family Follow-Up Study
Source: BMJ Open. 2017 Dec 21;7(12):e016949. doi: 10.1136/bmjopen-2017-016949 (PMC5778288; doi:10.1136/bmjopen-2017-016949)
Supplement: Supplementary file 4 [file bmjopen-2017-016949supp004.pdf]

Supplement 4. Mean[a] Anti-CMV IgG Titer Level[b] by Categories of Characteristics Obtained at Birth and Age 32 Among CMV seropositive individuals (N=1,051)

	Mean	(SE)	P-value[e]
**Characteristics obtained at birth**			
Maternal Religiosity			0.007
Ultra-Orthodox	14.34	(6.29)	
Orthodox	13.69	(7.09)	
Traditional	12.78	(6.34)	
Secular	12.37	(5.90)	
Paternal Lay-Leadership			0.06
Yes	14.12	(6.06)	
No	13.09	(6.52)	
Paternal Occupation Level			0.69
Low	13.11	(6.69)	
Middle	13.14	(6.53)	
High	13.48	(6.14)	
Maternal Education			0.26
<9 years	13.82	(7.02)	
9-12 years	13.07	(6.35)	
>12 years	13.00	(6.08)	
Number of Siblings			0.003
≤2	13.02	(6.48)	
3-4	12.92	(6.46)	
5+	13.72	(6.30)	
**Characteristics obtained at age 32**			
Religiosity			0.03
Ultra-Orthodox	13.70	(6.04)	
Orthodox	14.00	(7.08)	
Traditional	13.12	(6.45)	
Secular	12.52	(6.20)	
Occupation Level			0.06
Low	12.56	(6.12)	
Middle	13.90	(7.02)	
High	13.01	(6.12)	
Years of Education			0.32
≤ 12	13.53	(6.63)	
>12	13.10	(6.33)	
Parity			0.008
≤2	12.75	(6.44)	
3-4	13.73	(6.70)	
5+	14.07	(5.69)	

CMV = cytomegalovirus; SE = standard error

[a]Sex-adjusted

[b]square root transformed EU/ml

[c]Obtained through ANOVA
