# Supplementary material for: Associations of social environment, socioeconomic position and social mobility with immune response in young adults: the Jerusalem Perinatal Family Follow-Up Study
Source: BMJ Open. 2017 Dec 21;7(12):e016949. doi: 10.1136/bmjopen-2017-016949 (PMC5778288; doi:10.1136/bmjopen-2017-016949)
Supplement: Supplementary file 5 [file bmjopen-2017-016949supp005.pdf]

Supplement 5. Regression Coefficients and 95% CI of CMV in Relation to Household and Socioeconomic Components[b] in Childhood and Adulthood Among CMV Seropositive Population

	Continuous CMV[a]					
	Model 1[c]			Model 2[c]		
**Component[b]**	**Coef.[d]**	***P***	**95% CI**	**Coef.[d]**	***P***	**95% CI**
Household						
Childhood	-0.41	0.008	-0.71, -0.11	-0.03	0.91	-0.44, 0.39
Adulthood	-0.74	<0.001	-1.07, -0.42	-0.67	0.007	-1.15, -0.18
Socioeconomic						
Childhood	-0.23	0.24	-0.61, 0.15	-0.21	0.31	-0.63, 0.20
Adulthood	-0.01	0.94	-0.35, 0.38	-0.17	0.42	-0.25, 0.59

Coef. = coefficient; CI = confidence interval; CMV = cytomegalovirus

[a]Continuous square root transformed EU/ml

[b]Created using principal component analysis detailed in methods

[c]Model 1 includes each component separately. Model 2 includes all four components together in model. All models adjusted for sex, maternal and paternal age at offspring birth and maternal, paternal and offspring smoking

[d]Coefficient = change in anti-CMV IgG titer level per increase in one unit of component (distribution characteristics in supplement 3)
