# Supplementary material for: Associations of social environment, socioeconomic position and social mobility with immune response in young adults: the Jerusalem Perinatal Family Follow-Up Study
Source: BMJ Open. 2017 Dec 21;7(12):e016949. doi: 10.1136/bmjopen-2017-016949 (PMC5778288; doi:10.1136/bmjopen-2017-016949)
Supplement: Supplementary file 6 [file bmjopen-2017-016949supp006.pdf]

Supplement 6. Associations between social environment and socioeconomic factors with CMV antibody in total and CMV seropositive population

	Among Total Population						Among CMV Seropositive Only					
	Univariate Model[a]			Multivariate Model[b]			Univariate Model[a]			Multivariate Model[b]		
	B	P-value	95% CI	β	P-value	95% CI	B	P-value	95% CI	β	P-value	95% CI
**Characteristics at birth**												
Maternal education (yrs)[d]	-0.32	<0.001	-0.45, -0.19	-0.35	<0.001	-0.52, -0.19	-0.11	0.08	-0.23, 0.01	-0.22	0.007	-0.37, -0.06
Maternal religiosity[c]	2.28	<0.001	1.39, 3.17	-0.47	0.51	-1.89, 0.94	1.17	0.005	0.35, 1.99	-0.30	0.65	-1.59, 0.99
Paternal lay leadership[c]	2.40	<0.001	1.09, 3.71	-0.82	0.39	-2.70, 1.05	0.88	0.13	-0.26, 2.04	-0.47	0.59	-2.18, 1.24
Paternal SEP[d]	-0.09	0.46	-0.34, 0.15	0.16	0.31	-0.15, 0.48	0.12	0.31	-0.11, 0.35	0.22	0.15	-0.08, 0.54
Family size[d]	0.51	<0.001	0.34, 0.67	0.18	0.16	-0.07, 0.44	0.16	0.04	0.007, 0.30	0.01	0.91	-0.22, 0.25
**Characteristics at age 32**												
Education (yrs)[d]	-0.15	0.01	-0.27, -0.04	-0.07	0.36	-0.21, 0.08	0.01	0.81	-0.10, 0.12	0.10	0.18	-0.04, 0.24
Religiosity[c]	2.74	<0.001	1.89, 3.60	2.24	0.002	0.82, 3.66	1.55	<0.001	0.77, 2.34	1.88	0.004	0.60, 3.17
Current Family size[d]	0.79	<0.001	0.58, 1.00	0.22	0.15	-0.08, 0.52	0.28	0.005	0.08, 0.48	-0.13	0.37	-0.42, 0.16
Current SEP[d]	-0.01	0.92	-0.31, 0.28	0.09	0.58	-0.24, 0.43	0.20	0.15	-0.07, 0.48	0.08	0.64	-0.24, 0.39

[a]Univariate model includes each variable alone in model
[b]Multivariate model includes all variables together in one model + offspring sex, maternal and paternal age at childbirth, maternal and paternal smoking during pregnancy and offspring smoking in young-adulthood
[c]Treated as dichotomous variables
[d]Treated as continuous variables
[e]SEP in 6 levels
