# Supplementary material for: Associations of social environment, socioeconomic position and social mobility with immune response in young adults: the Jerusalem Perinatal Family Follow-Up Study
Source: BMJ Open. 2017 Dec 21;7(12):e016949. doi: 10.1136/bmjopen-2017-016949 (PMC5778288; doi:10.1136/bmjopen-2017-016949)
Supplement: Supplementary file 7 [file bmjopen-2017-016949supp007.pdf]

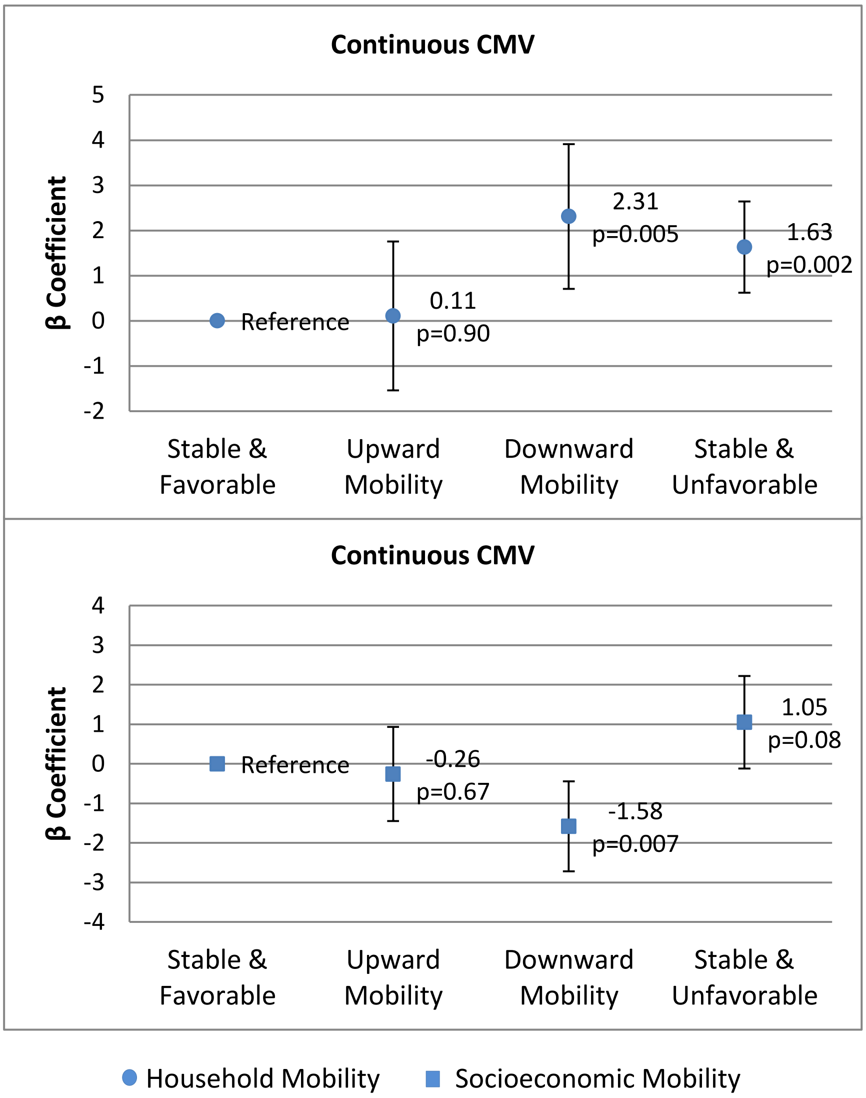

Supplement 7. Predicted mean anti-CMV IgG titer differences by household and socioeconomic mobility among the seropositive population. Predicted mean differences calculated via β coefficients from linear regression models adjusted for sex, maternal and paternal age at offspring birth and maternal, paternal and offspring smoking.
