# Supplementary material for: Associations of social environment, socioeconomic position and social mobility with immune response in young adults: the Jerusalem Perinatal Family Follow-Up Study
Source: BMJ Open. 2017 Dec 21;7(12):e016949. doi: 10.1136/bmjopen-2017-016949 (PMC5778288; doi:10.1136/bmjopen-2017-016949)
Supplement: Author's manuscript [file bmjopen-2017-016949.draft_revisions.pdf]

BMJ Open is committed to open peer review. As part of this commitment we make the peer review history of every article we publish publicly available.

When an article is published we post the peer reviewers' comments and the authors' responses online. We also post the versions of the paper that were used during peer review. These are the versions that the peer review comments apply to.

The versions of the paper that follow are the versions that were submitted during the peer review process. They are not the versions of record or the final published versions. They should not be cited or distributed as the published version of this manuscript.

BMJ Open is an open access journal and the full, final, typeset and author-corrected version of record of the manuscript is available on our site with no access controls, subscription charges or pay-per-view fees (http://bmjopen.bmj.com).

If you have any questions on BMJ Open's open peer review process please email editorial.bmjopen@bmj.com

BMJ Open

Socioeconomic mobility and immune response in young adults

Journal:	*BMJ Open*
Manuscript ID	bmjopen-2017-016949
Article Type:	Research
Date Submitted by the Author:	22-Mar-2017
Complete List of Authors:	Lawrence, Gabriella; The Hebrew University-Hadassah Medical Center, Jerusalem, Israel, The Braun School of Public Health<br>Friedlander, Yehiel; The Hebrew University of Jerusalem, The Braun School of Public Health and Community Medicine<br>Calderon-Margalit, Ronit; The Hebrew University of Jerusalem, Braun School of Public Health and Community Medicine<br>Enquobahrie, Daniel; University of Washington, Seattle, Department of Epidemiology, School of Public Health<br>Huang, Jonathan; McGill Unversity, Institute for Health and Social Policy<br>Tracy, Russell; University of Vermont<br>Manor, Orly; The Hebrew University of Jerusalem, Braun School of Public Health and Community Medicine<br>Siscovick, David; New York Academy of Medicine,<br>Hochner, Hagit; The Hebrew University-Hadassah Medical Center, Jerusalem, Israel, The Braun School of Public Health
**Primary Subject Heading**:	Epidemiology
Secondary Subject Heading:	Immunology (including allergy), Cardiovascular medicine, Sociology
Keywords:	EPIDEMIOLOGY, Immunology < BASIC SCIENCES, SOCIAL MEDICINE

SCHOLARONE™ Manuscripts

Socioeconomic mobility and immune response in young adults

List of Authors:

Gabriella M. Lawrence,[1] Yechiel Friedlander,[1] Ronit Calderon-Margalit,[1] Daniel A. Enquobahrie,[2,3] Jonathan Y. Huang,[3,4] Russell P. Tracy,[5] Orly Manor,[1] David S. Siscovick,[6] and Hagit Hochner[1]

[1]Braun School of Public Health, Hebrew University-Hadassah Medical Center, Jerusalem, Israel, [2]Cardiovascular Health Research Unit, [3]Department of Epidemiology, University of Washington, Seattle, WA, [4]Institute for Health and Social Policy, McGill University, Montreal, QC, [5]Departments of Pathology and Biochemistry, University of Vermont, Burlington, VT, USA [6]New York Academy of Medicine, New York, NY, USA

Correspondence to:
Hagit Hochner, PhD
Unit of Epidemiology, Hebrew University-Hadassah Braun School of Public Health
POB 12272, Jerusalem 91120, Israel
Phone: +972-2-6777502 Fax: +972-2-6431086
Email: hagith@ekmd.huji.ac.il

Word Count: 2,654

For peer review only

ABSTRACT

Objectives: Immune response to cytomegalovirus (CMV) impacts adult chronic disease. This study investigates associations of childhood and adulthood SEP and social mobility with CMV antibody response in young-adults.

Design: Historical prospective study design

Setting: Subcohort of all 17,003 births to residents of Jerusalem between 1974 and 1976.

Participants: Participants included 1319 young adults born in Jerusalem with extensive archival and follow-up data, including childhood and adulthood SEP-related factors and anti-CMV IgG titer levels and seroprevalence measured at age 32.

Main exposure and outcome measures: Principal component analysis (PCA) was used to transform SEP-related variables at two time points (childhood and adulthood) into two major scores reflecting household (e.g. number of siblings/children, religiosity) and socioeconomic (e.g. occupation, education) components. Based on these components, social mobility variables were created. Linear and Poisson regression models were used to investigate the associations of SEP components and mobility with antibody titers to CMV and seroprevalence, adjusted for confounders.

Results: Lower levels of household and socioeconomic components, reflecting worse SEP, in either childhood or adulthood were associated with higher CMV antibody levels and seropositivity at age 32. Compared to individuals with stable favorable components, CMV antibody titers and risk for seropositivity were higher in stable unfavorable household and socioeconomic components (household: $\beta=3.23, p<0.001$; RR=1.21, $p<0.001$; socioeconomic: $\beta=2.20, p=0.001$, RR=1.14, $p=0.01$), downward household mobility ($\beta=4.32$, $p<0.001$; RR=1.26, $p<0.001$) and upward socioeconomic mobility ($\beta=1.37$, $p=0.04$; RR=1.19, $p<0.001$).

Conclusions: Our study provides evidence that accumulating low SEP from childhood through adulthood and social mobility may compromise immune response in young adulthood.

For peer review only - http://bmjopen.bmj.com/site/about/guidelines.xhtml

Keywords: Immune System, Epidemiology, Socioeconomic Position, Social Mobility, Cytomegalovirus, Cardiovascular

Strengths and limitations of this study

- Cytomegalovirus (CMV) antibody as a biomarker for atherosclerosis is a novel addition to studies of cardio-metabolic risk
- Few studies have examined impact of socioeconomic position (SEP) on immune-response in adulthood
- This is a unique population-based birth cohort with rich archival and follow-up data from birth to young adulthood
- We used an aggregate view of SEP based on high-quality characterization of the socioeconomic environment in both childhood and adulthood to assess relationships of SEP in different time points in life as well as changes from childhood to adulthood with adult immune-response reflected by CMV titers
- Timing at initial seroconversion cannot be determined as only a single CMV titer measure was taken at young adulthood, also limiting precision in detection of lifetime viral load

review only

For peer review only - http://bmjopen.bmj.com/site/about/guidelines.xhtml

INTRODUCTION

Chronic inflammation is a known risk factor in cardiovascular disease, and infection-induced inflammation may be one of the causes of endothelial dysfunction leading to atherosclerosis[1]. Specifically, immune response to cytomegalovirus (CMV), a highly transmissible beta herpesvirus endemic throughout the world [2] and a biomarker of immune response [3,4], has been implicated in the development of atherosclerosis [3,5–7] and cardiovascular-related morbidity [8,9] and mortality[5,10–12]. Until recently, non-congenital CMV was not seen as clinically relevant in immunocompetent individuals due to the lack of overt clinical symptoms. However, accumulating evidence suggests that CMV antibody titer is associated with pro-inflammatory cytokines[3,4]; more memory T-cells are devoted to suppression of latent CMV [12,13] and fewer naïve T-cells are available to respond to new infections [12–14], leading to chronic immune activation and inflammation, a known risk-factor in cardiovascular disease. Despite the sizeable cellular response from the host, a characteristic unique to CMV, the virus adapts to the immune system efficiently. CMV, therefore, is never eliminated from the infected immunocompetent individual, in whom it causes a persistent asymptomatic infection [15,16].

Research suggests that early-life environments affect immune function over the life-course[17]. Studies show that early-life stress results in infection-related premature death, immunosuppression [18], impairment in components of cell-mediated immunity, reduction in antibody response to vaccination and elevated antibody level [17,19,20] during childhood and beyond. A life-course approach to health proposes that interaction of environment and biological factors – e.g. epigenetic programming - experienced throughout the life-course impact current and future health[21][21–23].

For peer review only - http://bmjopen.bmj.com/site/about/guidelines.xhtml

Associations between CMV and education, income [24], household crowding [2,25] and family size [26] further indicate that social factors influence immune response. Additionally, recent studies examining the impact of social mobility and socioeconomic gradients on adult health report varied results [27–30] . While upward mobility may attenuate the harmful effects of earlier disadvantage, and downward mobility is hypothesized to have a detrimental influence on health despite advantages at earlier ages, sustained exposure to low socioeconomic position (SEP) is most commonly associated with worse health [29–31]. Though associations between early-life socioeconomic factors and CMV have been demonstrated, the effect of social transitions from childhood to adulthood on CMV-response has not been investigated.

This study investigates associations between SEP in childhood and adulthood and social mobility from birth to 32 years with CMV-response in young-adults of the Jerusalem Perinatal Family Follow-up Study.

METHODS

The Jerusalem Perinatal Study (JPS) population-based cohort includes a subcohort of all 17 003 births to residents of Jerusalem between 1974 and 1976 [32,33]. Data consist of demographic and socioeconomic information, maternal medical conditions during current and previous pregnancies, and offspring birth-weight, abstracted from birth certificates or maternity ward logbooks. Additional information, including socioeconomic-related factors, gestational-age, smoking, pre-pregnancy and end of pregnancy weight, and gynecological history, was collected by interviewing mothers on first or second postpartum day. Detailed information on data collection is previously described [32,33].

For peer review only - http://bmjopen.bmj.com/site/about/guidelines.xhtml

The JPS Family Follow-Up study includes a sample of 1400 offspring from the 1974-1976 cohort interviewed and examined between 2007-2009 (mean age=32; range: 30-35 years) [34]. Briefly, sampling frame included singletons and term (gestational age ≥36 weeks) births without congenital malformations stratified by maternal pre-pregnancy BMI and birth-weight. Blood samples at fasting were taken and offspring IgG antibodies to CMV antigen were measured in plasma using indirect enzyme immunoassay with the Diamedix Immunosimplicity Is-CMV IgG Test Kit (Diamedex Corporation, 2008) at the University of Vermont. Further Test Kit details provided in supplement 1. Anti-CMV IgG titer levels were measured for 1319 offspring.

This study was approved by the Institutional Review Board of the Hadassah-Hebrew University Medical Center and by the University of Washington Human Subject Review Committee. All participants provided informed consent.

Study Variables

Outcome Variables

The primary outcome examined was offspring CMV-response measured through offspring antibody to CMV at age 32. Transformation (square-root) of anti-CMV IgG titer level (EU/ml) was performed due to right skewness and treated as a continuous variable. CMV seroprevalence (seropositivity ≥10 EU/ml) was examined as a secondary dichotomous outcome.

Explanatory Variables

The multi-ethnic society of Israel[35] provides an opportunity to examine SEP broadly, including examination of factors such as religiosity and family-size in addition to well-recognized SEP variables.

For peer review only - http://bmjopen.bmj.com/site/about/guidelines.xhtml

SEP-related explanatory variables were examined at birth and age 32, reflecting early (i.e., pre- and peri-natal periods) and young-adult life-course periods, respectively. Variables at childhood include (1)maternal religiosity (ultra-orthodox, orthodox, traditional, secular); (2)paternal lay-leadership (i.e. whether father was an ordained rabbi, dichotomous); (3)father's occupation (scale:1-6; 1-low, 6-high) as described by Corcoran[36]; (4)maternal education (years, continuous); and (5)number of siblings (continuous). Variables at adulthood include (1)offspring religiosity (ultra-orthodox, orthodox, traditional, secular); (2)offspring occupation (scale :1-6; 1-low, 6-high); (3)offspring education (years, continuous); and (4)parity at age 32 (continuous).

Potential confounders include offspring sex (dichotomous), maternal and paternal age at childbirth (years, continuous), maternal and paternal smoking during pregnancy and offspring smoking in young-adulthood (number of cigarettes smoked per day: 0, 1-10, 11-20, 21+).

Principal Component Analysis (PCA)

PCA [37] carried out separately for variables measured at childhood and adulthood, transformed SEP-related variables into two major components reflecting household (e.g. number of siblings/children, religiosity) and socioeconomic (e.g. occupation, education) environments. The two components together explained 74% of childhood and 75% of adulthood variance. Detailed information on component construction, variables included in PCA and distribution characteristics provided in supplement 2. Higher component score indicates improvement in household (i.e. fewer children/decreased religiosity) or socioeconomic (i.e. higher occupation level/years of education) factors.

Social Mobility

For peer review only - http://bmjopen.bmj.com/site/about/guidelines.xhtml

For peer review only

For both household and socioeconomic components, childhood to adulthood social mobility variables were created. Components were divided into tertiles and dichotomous variables were created for each component (lowest tertile = low; middle and high tertile = high). Four categories of childhood to adulthood transition were created separately for household and socioeconomic component mobility: stable favorable (high-high), upward mobility (low-high), downward mobility (high-low), and stable unfavorable (low-low).

Statistical Analyses

Analysis of variance model, adjusted for sex, was used to compare mean anti-CMV IgG titer level by categories of characteristics obtained at birth and age 32, principal component tertiles and social mobility categories. Linear regression models were used to investigate associations of childhood and adulthood household and socioeconomic components and social mobility with anti-CMV IgG titer level measured at 32. Multivariable models were fitted to assess associations between childhood and adulthood components, independently of each other, with anti-CMV IgG titer controlling for sex, parental ages at offspring birth and parental and offspring smoking. Similar multivariable models were fitted to assess associations between household and socioeconomic mobility with anti-CMV IgG titer. Poisson regression models with robust error variance were used to estimate relative risk of CMV seropositivity for each unit increase in component scores and among categories of social mobility variables.

Sex interactions with characteristics on CMV were assessed by introducing multiplicative terms (mppbmiXsex, GWGXsex, birth weightXsex) into the linear regression models.

For peer review only

All models used inverse probability weighting to account for stratified sampling. Analyses were carried out using Stata 12.0 (StataCorp, College Station, TX).

RESULTS

Parental and offspring characteristics obtained at birth and offspring characteristics at age 32, including anti-CMV IgG titer, CMV seroprevalence and social mobility are listed in Table 1. Mean anti-CMV IgG titer and seropositivity were greater among females than males. Overall, 79.7% of the study population was seropositive for CMV. For social mobility, 7.2% of the population was categorized as downwardly mobile in household component and 15.2% experienced downward socioeconomic-mobility; 7.2% and 16.9% experienced upward household and socioeconomic mobility respectively.

Table 1. Study Characteristics at Birth and Age 32[a] * (N=1319)

**Cytomegalovirus**		
Anti-CMV IgG titer level[b]		
Females	12.0	(7.9)
Males	9.5	(7.7)
Total	10.8	(7.9)
CMV Seropositivity[b] %		
Females	84.4	
Males	75.0	
Total	79.7	
**Characteristics obtained at birth**		
Maternal Religiosity %		
Ultra-Orthodox	18.4	
Orthodox	22.2	
Traditional	35.2	
Secular	24.2	
Paternal Lay Leadership %		
Yes	14.0	
No	86.0	
Paternal Occupation Level %		
Low	40.0	
Middle	23.3	

For peer review only - http://bmjopen.bmj.com/site/about/guidelines.xhtml

High	36.7	
Maternal Years of Education (years)	11.6	(3.4)
Number of Siblings %		
≤2	30.0	
3-4	39.5	
5+	30.5	
Maternal Age at Childbirth (years)	28.1	(5.6)
Paternal Age at Childbirth (years)	32.0	(6.9)
Maternal Smoking in Pregnancy %		
No	86.7	
Yes	13.3	
Paternal Smoking in Pregnancy %		
No	56.6	
Yes	43.4	
**Characteristics obtained at age 32**		
Religiosity %		
Ultra-Orthodox	20.5	
Orthodox	20.8	
Traditional	20.6	
Secular	38.1	
Occupation Level %		
Low	10.8	
Middle	33.2	
High	56.0	
Years of Education	15.5	(3.9)
Parity %		
≤2	62.6	
3-4	22.8	
5+	14.6	
Smoking %		
No	59.4	
Yes	40.6	
**Social Mobility**[d]		
Household[c] Mobility %		
Stable & Favorable	58.8	
Upward Mobility	7.1	
Downward Mobility	7.2	
Stable & Unfavorable	26.9	
Socioeconomic[c] Mobility %		
Stable & Favorable	49.5	
Upward Mobility	16.9	
Downward Mobility	15.2	
Stable & Unfavorable	18.4	

For peer review only

For peer review only - http://bmjopen.bmj.com/site/about/guidelines.xhtml

CMV = cytomegalovirus
[a]Values are expressed as mean (Standard Deviation) or percent
[b]obtained at age 32; square root transformed EU/ml; Seroprevalence = IgG titer level ≥10 EU/ml
[c]Household and Socioeconomic components created using principal component analysis detailed in methods; higher component score indicates improvement in household or socioeconomic factors
[d]Social mobility variables represent transition from childhood to adulthood using dichotomized household and socioeconomic components

For peer review only

Table 2 presents mean anti-CMV IgG titer level by categories of characteristics obtained at birth and age 32, household and socioeconomic components and social mobility. Mean anti-CMV IgG was higher among offspring born to ultra-orthodox mothers and fathers holding religious leadership positions. Offspring born to mothers with >12 years of education and those with ≤2 siblings had lower mean anti-CMV IgG titer. Similarly, ultra-orthodox offspring, those with ≤12 years of education and with more than 5 children had higher mean anti-CMV IgG titer level. Those in the lowest tertile of household and socioeconomic components in childhood or adulthood had higher mean anti-CMV IgG titer level. For both household and socioeconomic component mobility, those maintaining a stable favorable position had lower anti-CMV IgG titer level.

Table 2. Mean[a] Anti-CMV IgG Titer Level[b] by Categories of Characteristics Obtained at Birth and Age 32, Principal Components[c] and Household and Socioeconomic Mobility[d]

	**Mean**	**(SE)**	**P-value**[e]
**Characteristics obtained at birth**			
Maternal Religiosity			<.0001
Ultra-Orthodox	12.9	(0.52)	
Orthodox	11.4	(0.47)	
Traditional	10.5	(0.37)	
Secular	8.5	(0.43)	
Paternal Lay-Leadership			<.0001
Yes	13.5	(0.23)	
No	10.3	(0.58)	

Paternal Occupation Level			0.8
Low	10.4	(0.34)	
Middle	10.6	(0.44)	
High	10.6	(0.35)	
Maternal Education			0.003
<9 years	11.9	(0.47)	
9-12 years	10.8	(0.33)	
>12 years	9.9	(0.36)	
Number of Siblings			<.0001
≤2	9.4	(0.38)	
3-4	10.3	(0.34)	
5+	12.8	(0.40)	
**Characteristics obtained at age 32**			
Religiosity			<0.0001
Ultra-Orthodox	12.6	(0.48)	
Orthodox	12.2	(0.48)	
Traditional	11.0	(0.48)	
Secular	9.1	(0.34)	
Occupation Level			0.01
Low	11.4	(0.68)	
Middle	11.6	(0.39)	
High	10.2	(0.29)	
Years of Education			0.001
≤ 12	11.9	(0.42)	
>12	10.3	(0.25)	
Parity			<0.0001
≤2	9.6	(0.27)	
3-4	12.5	(0.45)	
5+	13.1	(0.57)	
**Household Components**			
Childhood			<0.0001
Tertile 1 (lowest)	12.0	(0.39)	
Tertile 2	9.5	(0.39)	
Tertile 3	10.5	(0.39)	
Adulthood			<0.0001
Tertile 1 (lowest)	12.8	(0.37)	
Tertile 2	10.0	(0.37)	
Tertile 3	9.6	(0.39)	
**Socioeconomic Components**			<0.0001
Childhood			
Tertile 1 (lowest)	11.9	(0.39)	
Tertile 2	10.7	(0.40)	
Tertile 3	9.4	(0.39)	
Adulthood			<0.0001

For peer review only - http://bmjopen.bmj.com/site/about/guidelines.xhtml

Tertile 1 (lowest)	12.4	(0.38)	
Tertile 2	11.0	(0.38)	
Tertile 3	9.1	(0.37)	
**Household Mobility**			<0.0001
Stable & favorable	9.7	(0.29)	
Upward mobility	10.5	(0.85)	
Downward mobility	12.9	(0.88)	
Stable & unfavorable	12.5	(0.45)	
**Socioeconomic Mobility**			<0.0001
Stable & favorable	9.5	(0.32)	
Upward mobility	10.9	(0.56)	
Downward mobility	11.7	(0.58)	
Stable & unfavorable	13.1	(0.54)	

CMV = cytomegalovirus; SE = standard error

[a]Sex-adjusted

[b]square root transformed EU/ml

[c] Components created using principal component analysis detailed in methods; higher component score indicates improvement in household or socioeconomic factors

[d] Social mobility variables represent transition from childhood to adulthood using dichotomized household and socioeconomic components

[e]Obtained through ANOVA

For peer review only

In order to model associations and adjust for additional covariates (i.e. offspring sex, parental ages at birth and parental and offspring smoking), we investigated associations between household and socioeconomic components with anti-CMV IgG titer using linear regression models and with CMV seroprevalence using Poisson regression models presented in Table 3. Lower levels of household and socioeconomic components in childhood and adulthood, reflecting worse SEP, were associated with higher CMV-response at age 32 (Table 3, continuous CMV Model 1) and increased CMV seropositivity (Table 3, seroprevalence Model 1). When childhood and adulthood characteristics were included together in one model (Table 3, continuous CMV Model 2), only adulthood household and childhood socioeconomic

components were associated with anti-CMV IgG titer (β= -1.02, p<0.001 and β= -0.74, p=0.001, respectively).

Table 3. Regression Coefficients, Relative Risks and 95% CI of CMV in Relation to Household and Socioeconomic Components[c] in Childhood and Adulthood

	Continuous CMV[a]						Seroprevalence[b]					
	Model 1[d]			Model 2[d]			Model 1[d]			Model 2[d]		
**Component**[c]	**Coef.**[d]	***P***	**95% CI**	**Coef.**[d]	***P***	**95% CI**	**RR**[d]	***P***	**95% CI**	**RR**[d]	***P***	**95% CI**
Household												
Childhood	-0.78	<0.001	-1.11,-0.45	-0.07	0.76	-0.53,0.39	0.96	0.001	0.94,0.98	1.00	0.86	0.97,1.04
Adulthood	-1.25	<0.001	-1.61,-0.90	-1.02	<0.001	-1.55,-0.49	0.94	<0.001	0.91,0.97	0.95	0.02	0.91,0.99
Socioeconomic												
Childhood	-1.04	<0.001	-1.43,-0.65	-0.74	0.001	-1.17,-0.31	0.91	<0.001	0.88,0.95	0.94	0.001	0.90,0.98
Adulthood	-0.71	<0.001	-1.09,-0.33	-0.39	0.08	-0.82,0.04	0.93	<0.001	0.90,0.97	0.95	0.02	0.92,0.99

Coef. = coefficient; CI = confidence interval; CMV = cytomegalovirus; RR = relative risk
[a]continuous square root transformed EU/ml
[b] Anti-CMV IgG titer level ≥10 EU/ml
[c] Created using principal component analysis detailed in methods; Higher component score indicates improvement in household or socioeconomic factors.
[d]Model 1 includes each component separately. Model 2 includes all four components together in model. All models adjusted for sex, maternal and paternal age at offspring birth and maternal, paternal and offspring smoking
[d]Coefficient = change in anti-CMV IgG titer level per increase in one unit of component (distribution characteristics in supplement 2); RR = change in risk estimate of CMV seropositivity per increase in one unit of component – calculated by Poisson regression with robust error variance

We also investigated associations between household and socioeconomic component-mobility and anti-CMV IgG titer, adjusted for sex, parental ages at offspring birth and parental and offspring smoking (Figure 1). Compared to individuals with stable favorable household components, mean anti-CMV IgG levels were higher in those who maintained unfavorable household components from childhood to adulthood (β=3.23, p<0.001) and those who transitioned from a favorable to unfavorable position (i.e. downward

For peer review only

mobility) (β=4.32, p<0.001). Those who maintained a stable unfavorable socioeconomic component position had increased anti-CMV titer level compared to those in the stable favorable position (β=2.20, p=0.001) as did those in the upward mobility category (β=1.37, p=0.04). A similar pattern of associations was seen when investigating associations between mobility and CMV seroprevalence (Figure 1).

Discussion

This study demonstrated that lower levels of household and socioeconomic components in childhood and adulthood, reflecting worse SEP, were associated with higher CMV-response at age 32. Additionally, maintaining a stable unfavorable position from childhood to adulthood was associated with higher CMV-response compared to the stable favorable position. Our study also provides some evidence that social mobility transitions from childhood to adulthood (i.e. upward and downward mobility) may be associated with CMV-response.

Life-Course Models

Life-course models explaining the impact of SEP on adult health proposed in the literature include the critical period model, emphasizing timing of exposure[38,39], the social mobility model, focusing on transitions across the life-course[38,39], and the accumulation model, emphasizing duration of exposures[38,39]. As per the critical period model, our findings that lower childhood and adulthood household and socioeconomic components are associated with higher CMV-response in early adulthood are in accordance with other studies[17,40]. Our study also adds to the social mobility model, though unlike others[27,28,41], we found that upward socioeconomic mobility was associated with higher CMV titer and higher risk for CMV

For peer review only

seropositivity. Possibly, as others, including JPS analyses, have found[42], mechanisms similar to those in Gluckman's "Mismatch hypothesis” are at play – in which early environment induces long-term cardiovascular effects if there is a “mismatch” (including improvement) between early developmental environment and subsequent environment in later life. Household downward mobility, as in other cohort-based research[30], was associated with increased CMV IgG titer levels and increased risk for CMV seropositivity. Our study is the first to investigate social mobility associations with young-adult CMV-response.

Research has indicated that sustained exposure to low SEP has been most commonly associated with worse health[27,29–31,38,41]. Our study supports the detrimental impact of accumulation of lower SEP over the life-course, as we found increased anti-CMV IgG titer and risk for CMV seropositivity among those in both the household and socioeconomic stable unfavorable group as compared to stable favorable.

Multidimensionality of Exposure

The associations of social mobility with young-adult CMV-response differ somewhat in household and socioeconomic components. While household downward mobility was associated with increased CMV-response, upward socioeconomic mobility seemingly had a harmful impact on immune response at age 32. These findings highlight the multidimensionality of socioeconomic measurement and the varying contribution of different socioeconomic dimensions through the life-course[43]. The PCA used in our analyses may provide a more aggregate characterization of the socioeconomic experience/environment, while demonstrating that various socioeconomic factors may indeed represent different

For peer review only - http://bmjopen.bmj.com/site/about/guidelines.xhtml

domains. To further highlight this point, household upward or downward mobility comprised only 14.3% of the study population while socioeconomic mobility was seen among 31.1%, possibly indicating that the household component is more stable than the socioeconomic component. Possibly, people tend to maintain a level of religiosity similar to that with which they were raised[44] whereas socioeconomic transitions from parents to offspring vary depending on culture, environment, psychological elements and geographic residence[45].

Epigenetic Mechanism

Previous studies found that participants originating from low and high SEP have different patterns of methylation[46]. Additionally, associations have been found between methylation and chronic inflammation and a previous analysis of JPS demonstrated an *HSD11B2*–cardiometabolic risk association[23]. Epigenetic changes in response to environment may play an important role in the development of adult diseases[47]. Further studies that investigate an epigenetic role in the SEP–CMV associations may provide evidence for mechanisms that explain these associations.

Study Strengths and Limitations

One of the major strengths of this study is the novel use of CMV antibody, emerging as a potential biomarker for atherosclerosis and cardiometabolic risk, as well as the use of continuous CMV antibody in addition to CMV seropositivity as a study outcome. Few studies have examined CMV antibody titer on a continuous scale, despite potential epidemiological significance of such an outcome. Additionally, very few studies have had the ability to examine the impact of SEP and social mobility on immune-response in young adulthood. The

For peer review only - http://bmjopen.bmj.com/site/about/guidelines.xhtml

combination of high-quality detailed records of perinatal maternal and offspring characteristics and long-term follow-up data at age 32, improved the characterization of the socioeconomic environment surrounding pregnancy, childhood and adulthood, and allowed analyses to examine changes from childhood to adulthood. Estimating different components of the socioeconomic environment and trajectory by PCA was another novel approach, enabling us to capture common variance and providing an aggregate view of the socioeconomic experience/environment, rather than limiting analyses to individual SEP proxies.

Our study has several limitations. First, it includes only a sample of offspring from the original 1974-1976 JPS cohort invited to participate in the follow-up study. However, using a stratified sampling approach and over-sampling ends of the distribution ensured that offspring with a range of fetal and early-life characteristics were included in our study. Second, examining CMV titer on a continuous scale using qualitative ELISA assay limits clinical interpretation of findings. However, our findings using CMV seropositivity, a clinically relevant diagnosis, support the associations found with CMV antibody titer, and substantiate the use of continuous CMV antibody in future epidemiological studies. Importantly, this specific assay was successfully used in other cohorts, such as the Multi-Ethnic Study of Atherosclerosis[12]. Additionally, data consists of a single CMV titer measure for each participant. Verification of titer level from additional tests or other sources was not available, and timing/age at initial seroconversion is unknown. However, research has indicated that CMV antibody titer remains relatively stable over time[48,49], and seroprevalence rates in our population fall in the range of prevalence rates in similar populations[2,15,16,50].

For peer review only - http://bmjopen.bmj.com/site/about/guidelines.xhtml

For peer review only

Conclusions

Our study extends accumulating evidence of relationships between socioeconomic factors in childhood and adulthood with adult immune response. Additionally, our study provides evidence that accumulating low SEP from childhood through adulthood and social mobility may compromise immune response in young adulthood. Further studies should explore mechanisms underlying the relationships between childhood environments and social mobility with immunity in adulthood. A better understanding of these relationships may lead to new approaches to counteract effects of low-SEP environments through the life-course and improve immune response in high-risk young-adults.

For peer review only

For peer review only - http://bmjopen.bmj.com/site/about/guidelines.xhtml

REFERENCES

1 Ross R. Inflammation or Atherogenesis. *N Engl J Med* 1999;**340**:115–26. doi:10.1056/NEJM199901143400207

2 Bate SL, Dollard SC, Cannon MJ. Cytomegalovirus seroprevalence in the United States: the national health and nutrition examination surveys, 1988-2004. *Clin Infect Dis* 2010;**50**:1439–47. doi:10.1086/652438

3 Nazmi A, Diez-Roux A V, Jenny NS, *et al.* The influence of persistent pathogens on circulating levels of inflammatory markers: a cross-sectional analysis from the Multi-Ethnic Study of Atherosclerosis. *BMC Public Health* 2010;**10**:706. doi:10.1186/1471-2458-10-706

4 Marashi SM, Raeiszadeh M, Enright V, *et al.* Influence of cytomegalovirus infection on immune cell phenotypes in patients with common variable immunodeficiency. *J Allergy Clin Immunol* 2012;**129**:1349–1356.e3. doi:10.1016/j.jaci.2012.02.011

5 Simanek AM, Dowd JB, Pawelec G, *et al.* Seropositivity to cytomegalovirus, inflammation, all-cause and cardiovascular disease-related mortality in the United States. *PLoS One* 2011;**6**:e16103. doi:10.1371/journal.pone.0016103

6 Zhu J, Quyyumi A a, Norman JE, *et al.* Cytomegalovirus in the pathogenesis of atherosclerosis. *J Am Coll Cardiol* 1999;**34**:1738–43. doi:10.1016/S0735-1097(99)00410-6

7 Blum a., Peleg a., Weinberg M. Anti-cytomegalovirus (CMV) IgG antibody titer in patients with risk factors to atherosclerosis. *Clin Exp Med* 2003;**3**:157–60. doi:10.1007/s10238-003-0019-7

8 Ji Y-N, An L, Zhan P, *et al.* Cytomegalovirus infection and coronary heart disease risk: a meta-analysis. *Mol Biol Rep* 2012;**39**:6537–46. doi:10.1007/s11033-012-1482-6

9 Zhang M, Yang Y, Yang X, *et al.* Human cytomegalovirus infection is a novel etiology for essential hypertension. *Med Hypotheses* 2011;**76**:682–4. doi:10.1016/j.mehy.2011.01.032

10 Roberts ET, Haan MN, Dowd JB, *et al.* Cytomegalovirus antibody levels, inflammation, and mortality among elderly Latinos over 9 years of follow-up. *Am J Epidemiol* 2010;**172**:363–71. doi:10.1093/aje/kwq177

11 Strandberg TE, Pitkala KH, Tilvis RS. Cytomegalovirus antibody level and mortality among community-dwelling older adults with stable cardiovascular disease. *JAMA* 2009;**301**:380–2. doi:10.1001/jama.2009.4

12 Tracy RP, Doyle MF, Olson NC, *et al.* T-helper type 1 bias in healthy people is associated with cytomegalovirus serology and atherosclerosis: the Multi-Ethnic Study of Atherosclerosis. *J Am Heart Assoc* 2013;**2**:e000117. doi:10.1161/JAHA.113.000117

13 Olson NC, Doyle MF, Jenny NS, *et al.* Decreased naive and increased memory CD4(+) T cells are associated with subclinical atherosclerosis: the multi-ethnic study of atherosclerosis. *PLoS One* 2013;**8**:e71498. doi:10.1371/journal.pone.0071498

14 Zhu J. Discordant cellular and humoral immune responses to cytomegalovirus infection in

For peer review only - http://bmjopen.bmj.com/site/about/guidelines.xhtml

healthy blood donors: existence of a Th1-type dominant response. *Int Immunol* 2001;**13**:785–90. doi:10.1093/intimm/13.6.785

15 La Rosa C, Diamond DJ. The immune response to human CMV. *Future Virol* 2012;**7**:279–93. doi:10.2217/fvl.12.8

16 Levine H, Balicer RD, Rozhavski V, *et al.* Seroepidemiology of Epstein-Barr virus and cytomegalovirus among Israeli male young adults. *Ann Epidemiol* 2012;**22**:783–8. doi:10.1016/j.annepidem.2012.06.099

17 McDade TW. Life history, maintenance, and the early origins of immune function. *Am J Hum Biol* 2005;**17**:81–94. doi:10.1002/ajhb.20095

18 Moore SE, Cole TJ, Collinson AC, *et al.* Prenatal or early postnatal events predict infectious deaths in young adulthood in rural Africa. *Int J Epidemiol* 1999;**28**:1088–95.

19 Shirtcliff EA, Coe CL, Pollak SD. Early childhood stress is associated with elevated antibody levels to herpes simplex virus type 1. *Proc Natl Acad Sci U S A* 2009;**106**:2963–7. doi:10.1073/pnas.0806660106

20 McDade TW, Stallings JF, Angold A, *et al.* Epstein-Barr virus antibodies in whole blood spots: a minimally invasive method for assessing an aspect of cell-mediated immunity. *Psychosom Med* 2000;**62**:560–7.

21 Power C, Atherton K, Strachan DP, *et al.* Life-course influences on health in British adults: effects of socio-economic position in childhood and adulthood. *Int J Epidemiol* 2007;**36**:532–9. doi:10.1093/ije/dyl310

22 Borghol N, Suderman M, McArdle W, *et al.* Associations with early-life socio-economic position in adult DNA methylation. *Int J Epidemiol* 2012;**41**:62–74. doi:10.1093/ije/dyr147

23 Huang JY, Gavin AR, Richardson TS, *et al.* Accounting for life course exposures in epigenetic biomarker association studies: Early life socioeconomic position, candidate gene DNA methylation, and adult cardiometabolic risk. *Am J Epidemiol* 2016;**in-press**.

24 Dowd JB, Aiello AE. Socioeconomic differentials in immune response. *Epidemiology* 2009;**20**:902–8. doi:10.1097/EDE.0b013e3181bb5302

25 Jansen MAE, van den Heuvel D, Bouthoorn SH, *et al.* Determinants of Ethnic Differences in Cytomegalovirus, Epstein-Barr Virus, and Herpes Simplex Virus Type 1 Seroprevalence in Childhood. *J Pediatr* 2016;**170**:126–34. doi:10.1016/j.jpeds.2015.11.014

26 Shigemi D, Yamaguchi S, Otsuka T, *et al.* Seroprevalence of cytomegalovirus IgG antibodies among pregnant women in Japan from 2009-2014. *Am J Infect Control* 2015;**43**:1218–21. doi:10.1016/j.ajic.2015.06.026

27 Johnson-Lawrence V, Kaplan G, Galea S. Socioeconomic mobility in adulthood and cardiovascular disease mortality. *Ann Epidemiol* 2013;**23**:167–71. doi:10.1016/j.annepidem.2013.02.004

28 Aitsi-Selmi A, Batty GD, Barbieri MA, *et al.* Childhood socioeconomic position, adult socioeconomic position and social mobility in relation to markers of adiposity in early

For peer review only

For peer review only - http://bmjopen.bmj.com/site/about/guidelines.xhtml

adulthood: evidence of differential effects by gender in the 1978/79 Ribeirao Preto cohort study. *Int J Obes (Lond)* 2013;**37**:439–47. doi:10.1038/ijo.2012.64

29 Heraclides A, Brunner E. Social mobility and social accumulation across the life course in relation to adult overweight and obesity: the Whitehall II study. *J Epidemiol Community Health* 2010;**64**:714–9. doi:10.1136/jech.2009.087692

30 Smith BT, Lynch JW, Fox CS, *et al.* Life-course socioeconomic position and type 2 diabetes mellitus: The Framingham Offspring Study. *Am J Epidemiol* 2011;**173**:438–47. doi:10.1093/aje/kwq379

31 Halonen JI, Stenholm S, Pentti J, *et al.* Childhood Psychosocial Adversity and Adult Neighborhood Disadvantage as Predictors of Cardiovascular Disease: A Cohort Study. *Circulation* 2015;**132**:371–9. doi:10.1161/CIRCULATIONAHA.115.015392

32 Davies AM, Prywes R, Tzur B, *et al.* The Jerusalem perinatal study. 1. Design and organization of a continuing, community-based, record-linked survey. *Isr J Med Sci* 1969;**5**:1095–106.

33 Harlap S, Davies A, Deutsch L, *et al.* The Jerusalem Perinatal Study cohort, 1964-2005: methods and a review of the main results. *Paediatr Perinat Epidemiol* 2007;**21**:256–73. doi:10.1111/j.1365-3016.2007.00799.x.The

34 Lawrence GM, Siscovick DS, Calderon-Margalit R, *et al.* Cohort Profile: The Jerusalem Perinatal Family Follow-Up Study. *Int J Epidemiol* 2016;**45**:343–52. doi:10.1093/ije/dyv120

35 Haj M Al. *Immigration and Ethnic Formation in a Deeply Divided Society: The Case of the 1990s Immigrants from the Former Soviet Union in Israel*. BRILL 2004.

36 Corcoran C, Perrin M, Harlap S, *et al.* Effect of socioeconomic status and parents' education at birth on risk of schizophrenia in offspring. Soc. Psychiatry Psychiatr. Epidemiol. 2009;**44**:265–71. doi:10.1007/s00127-008-0439-5

37 Stevens J. *Applied multivariate statistics for the social sciences*. L. Erlbaum Associates Inc. 1986.

38 Pollitt RA, Rose KM, Kaufman JS. Evaluating the evidence for models of life course socioeconomic factors and cardiovascular outcomes: a systematic review. *BMC Public Health* 2005;**5**:7. doi:10.1186/1471-2458-5-7

39 Kuh D, Ben-Shlomo Y, Lynch J, *et al.* Life course epidemiology. *J Epidemiol Community Health* 2003;**57**:778–83.

40 Demakakos P, Marmot M, Steptoe A. Socioeconomic position and the incidence of type 2 diabetes: The ELSA study. *Eur J Epidemiol* 2012;**27**:367–78. doi:10.1007/s10654-012-9688-4

41 Albrecht SS, Gordon-Larsen P. Socioeconomic gradients in body mass index (BMI) in US immigrants during the transition to adulthood: examining the roles of parental education and intergenerational educational mobility. *J Epidemiol Community Health* 2014;**68**:842–8. doi:10.1136/jech-2014-203814

For peer review only

42 Savitsky B, Manor O, Friedlander Y, *et al.* Associations of socioeconomic position in childhood and young adulthood with cardiometabolic risk factors: the Jerusalem Perinatal Family Follow-Up Study. *J Epidemiol Community Health* 2017;**71**:43–51. doi:10.1136/jech-2014-204323

43 Cutler DM, Lleras-Muney A VT. Socioeconomic status and health: Dimensions and mechanisms. *Oxford Handb Heal Econ* 2011;:124–163.

44 Ashton MC. *Individual Differences and Personality*. Academic Press 2013.

45 *From Parents to Children: The Intergenerational Transmission of Advantage*. Russell Sage Foundation 2012.

46 Lam LL, Emberly E, Fraser HB, *et al.* Factors underlying variable DNA methylation in a human community cohort. *Proc Natl Acad Sci U S A* 2012;**109 Suppl**:17253–60. doi:10.1073/pnas.1121249109

47 Gilbert ER, Liu D. Epigenetics: the missing link to understanding β-cell dysfunction in the pathogenesis of type 2 diabetes. *Epigenetics* 2012;**7**:841–52. doi:10.4161/epi.21238

48 Zanghellini F, Boppana SB, Emery VC, *et al.* Asymptomatic primary cytomegalovirus infection: virologic and immunologic features. *J Infect Dis* 1999;**180**:702–7. doi:10.1086/314939

49 Miller MS, Gardner TJ, Krammer F, *et al.* Neutralizing antibodies against previously encountered influenza virus strains increase over time: a longitudinal analysis. *Sci Transl Med* 2013;**5**:198ra107. doi:10.1126/scitranslmed.3006637

50 Cannon MJ, Schmid DS, Hyde TB. Review of cytomegalovirus seroprevalence and demographic characteristics associated with infection. *Rev Med Virol* 2010;**20**:202–13. doi:10.1002/rmv

Contributors: DSS, HH and YF conceived and designed the study. HH designed data collection tools and developed protocols.GML designed the analysis plan, conducted the analysis and wrote the manuscript with guidance from YF and HH. DSS and RPT advised on the clinical context of the study and analyses.OM, RC-M, DAE and JYH advised on data analysis and interpretation of findings. All authors contributed intellectually to the manuscript, reviewed and edited the manuscript and approved the final version.

Funding: This work was financially supported by the National Institutes of Health [grant numbers R01HL088884 and K01HL103174] and the Israeli Science Foundation [grant numbers 1252/07 and 552/12].

Disclaimer: The founders had no role in the design, analysis or writing of this article

Competing interests: None declared

Informed Consent: Informed consent was obtained from all individual participants included in the study.

For peer review only - http://bmjopen.bmj.com/site/about/guidelines.xhtml

Ethical Approval: This study was approved by the Institutional Review Board of the Hadassah-Hebrew University Medical Center and by the University of Washington Human Subject Review Committee.

Data sharing statement: Data are available on request. Email Dr. Hagit Hochner: hagith@ekmd.huji.ac.il

For peer review only

For peer review only - http://bmjopen.bmj.com/site/about/guidelines.xhtml

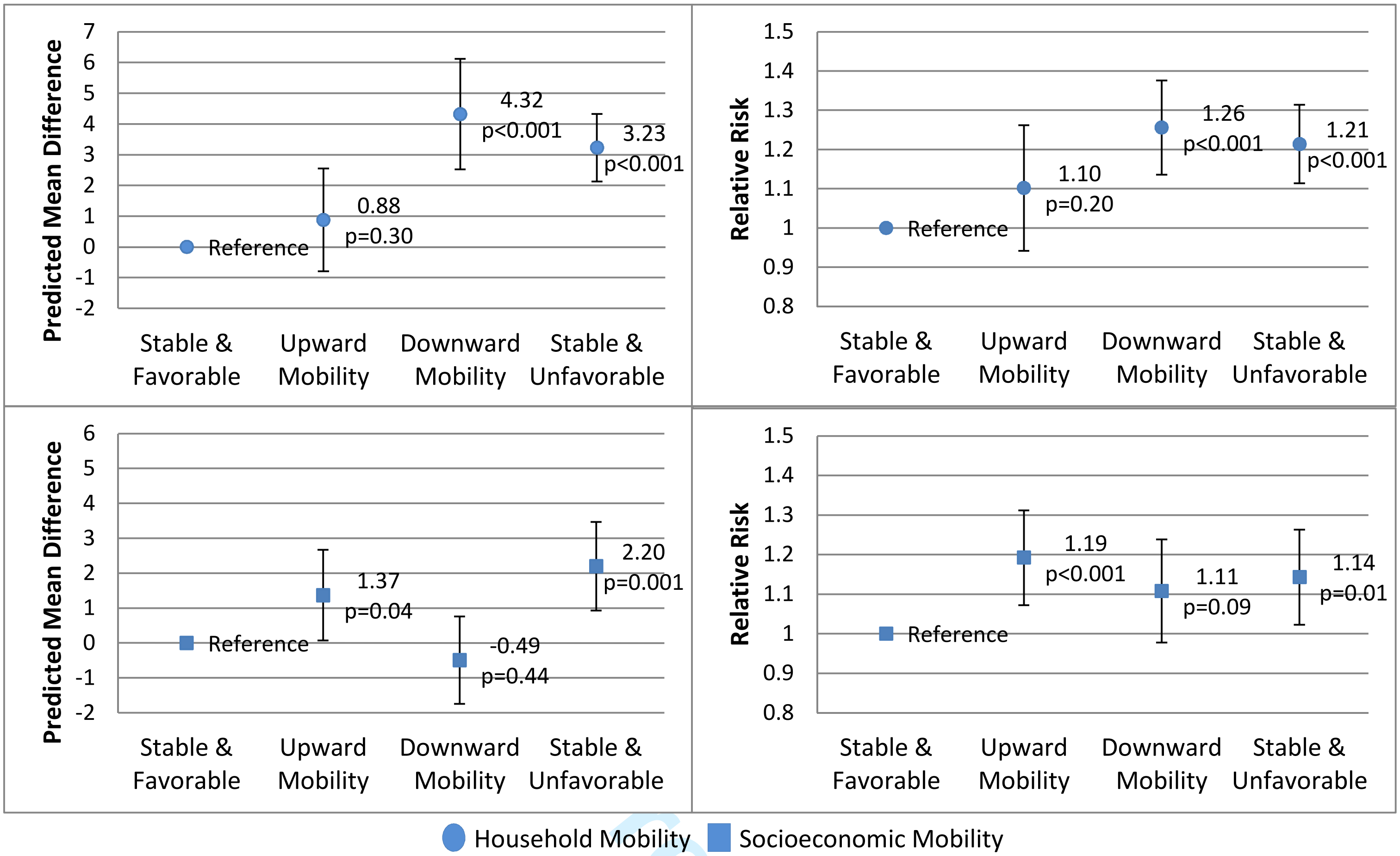

Fig. Predicted mean anti-CMV IgG titer differences and CMV seropositivity risk by household and socioeconomic mobility. Predicted mean differences calculated via β coefficients from linear regression models adjusted for sex, maternal and paternal age at offspring birth and maternal, paternal and offspring smoking. Relative risk estimation by Poisson regression with robust error variance models adjusted for sex, maternal and paternal age at offspring birth and maternal, paternal and offspring smoking

Supplement 1. Diamedix Immunosimplicity Is-CMV IgG Test Kit

(Diamedex Corporation, 2008)

The Test Kit is intended for the qualitative and semi-quantitative detection of antibodies to CMV antigen. Compared to currently used testing methods in three different United States-based laboratories, the sensitivity and specificity of the Diamedix Kit ranged from 99.3%-100.0% and 87.7%-100.0% respectively, and overall agreement ranged from 97.0%-99.0%. Seroprevalence results detected using this kit were in agreement with prevalence rates reported in United States blood donor populations.

Diamedix. CMV IgG: Enzyme Immunoassay Test Kit. 2008;3–6.

Childhood to adulthood socioeconomic position and immune response in young adults: The Jerusalem Perinatal Study, European Journal of Epidemiology, Gabriella M. Lawrence, Yechiel Friedlander, Ronit Calderon-Margalit, Daniel A. Enquobahrie, Jonathan Y. Huang, Russell P. Tracy, Orly Manor, David S. Siscovick,Hagit Hochner

Correspondence to:
Gabriella Lawrence, MPH, PhDc
Unit of Epidemiology, Hebrew University-Hadassah Braun School of Public Health
Email: gabriellalawrence@gmail.com

For peer review only

Supplement 2. Principal Component Analysis Loadings

Childhood			Adulthood		
	Component Loadings			Component Loadings	
Variables Included	Household	Socioeconomic	Variables Included	Household	Socioeconomic
Paternal Occupation	0.112	0.656	Offspring Occupation	-0.111	0.719
Maternal Education	-0.132	0.723	Offspring Education	0.115	0.690
Maternal Religiosity	0.538	0.048	Offspring Religiosity	0.695	0.056
Paternal Lay Leadership	0.550	0.119			
Number of Siblings	0.616	-0.180	Offspring Parity	0.701	-0.065
Variance Explained	74%		Variance Explained	75%	

Principal Component Score Distribution

	Mean	SD	Minimum	Maximum
**Household Components**				
Childhood	-0.003	1.49	-4.63	1.86
Adulthood	-0.008	1.27	-4.05	2.08
**Socioeconomic Components**				
Childhood	0.04	1.21	-4.14	3.52
Adulthood	0.06	1.15	-3.66	3.02

Childhood to adulthood socioeconomic position and immune response in young adults:
The Jerusalem Perinatal Study, European Journal of Epidemiology, Gabriella M. Lawrence, Yechiel Friedlander, Ronit Calderon-Margalit, Daniel A. Enquobahrie, Jonathan Y. Huang, Russell P. Tracy, Orly Manor, David S. Siscovick, Hagit Hochner

Correspondence to:
Gabriella Lawrence, MPH, PhDc
Unit of Epidemiology, Hebrew University-Hadassah Braun School of Public Health
Email: gabriellalawrence@gmail.com

For peer review only

STROBE Statement—checklist of items that should be included in reports of observational studies

	Item No.	Recommendation	Page No.
**Title and abstract**	1	(*a*) Indicate the study's design with a commonly used term in the title or the abstract	1
		(*b*) Provide in the abstract an informative and balanced summary of what was done and what was found	1
**Introduction**			
Background/rationale	2	Explain the scientific background and rationale for the investigation being reported	4
Objectives	3	State specific objectives, including any prespecified hypotheses	5
**Methods**			
Study design	4	Present key elements of study design early in the paper	5
Setting	5	Describe the setting, locations, and relevant dates, including periods of recruitment, exposure, follow-up, and data collection	5
Participants	6	(*a*) *Cohort study*—Give the eligibility criteria, and the sources and methods of selection of participants. Describe methods of follow-up<br>*Case-control study*—Give the eligibility criteria, and the sources and methods of case ascertainment and control selection. Give the rationale for the choice of cases and controls<br>*Cross-sectional study*—Give the eligibility criteria, and the sources and methods of selection of participants	5
		(*b*) *Cohort study*—For matched studies, give matching criteria and number of exposed and unexposed<br>*Case-control study*—For matched studies, give matching criteria and the number of controls per case	NA
Variables	7	Clearly define all outcomes, exposures, predictors, potential confounders, and effect modifiers. Give diagnostic criteria, if applicable	6-7
Data sources/ measurement	8*	For each variable of interest, give sources of data and details of methods of assessment (measurement). Describe comparability of assessment methods if there is more than one group	*6-7*
Bias	9	Describe any efforts to address potential sources of bias	
Study size	10	Explain how the study size was arrived at	6

Continued on next page

For peer review only - http://bmjopen.bmj.com/site/about/guidelines.xhtml

Quantitative variables	11	Explain how quantitative variables were handled in the analyses. If applicable, describe which groupings were chosen and why	6-7
Statistical methods	12	(*a*) Describe all statistical methods, including those used to control for confounding	7-8
		(*b*) Describe any methods used to examine subgroups and interactions	7-8
		(*c*) Explain how missing data were addressed	7-8
		(*d*) *Cohort study*—If applicable, explain how loss to follow-up was addressed<br>*Case-control study*—If applicable, explain how matching of cases and controls was addressed<br>*Cross-sectional study*—If applicable, describe analytical methods taking account of sampling strategy	
		(*e*) Describe any sensitivity analyses	
**Results**			
Participants	13*	(a) Report numbers of individuals at each stage of study—eg numbers potentially eligible, examined for eligibility, confirmed eligible, included in the study, completing follow-up, and analysed	Previously reported and referenced
		(b) Give reasons for non-participation at each stage	
		(c) Consider use of a flow diagram	
Descriptive data	14*	(a) Give characteristics of study participants (eg demographic, clinical, social) and information on exposures and potential confounders	8-9
		(b) Indicate number of participants with missing data for each variable of interest	Table 1
		(c) *Cohort study*—Summarise follow-up time (eg, average and total amount)	
Outcome data	15*	*Cohort study*—Report numbers of outcome events or summary measures over time	*8 and Table 1*
		Case-control study—Report numbers in each exposure category, or summary measures of exposure	
		Cross-sectional study—Report numbers of outcome events or summary measures	
Main results	16	(*a*) Give unadjusted estimates and, if applicable, confounder-adjusted estimates and their precision (eg, 95% confidence interval). Make clear which confounders were adjusted for and why they were included	Table 2 & 3
		(*b*) Report category boundaries when continuous variables were categorized	
		(*c*) If relevant, consider translating estimates of relative risk into absolute risk for a meaningful time period	

Continued on next page

For peer review only - http://bmjopen.bmj.com/site/about/guidelines.xhtml

Other analyses	17	Report other analyses done—eg analyses of subgroups and interactions, and sensitivity analyses	12-14
**Discussion**			
Key results	18	Summarise key results with reference to study objectives	15
Limitations	19	Discuss limitations of the study, taking into account sources of potential bias or imprecision. Discuss both direction and magnitude of any potential bias	15-16
Interpretation	20	Give a cautious overall interpretation of results considering objectives, limitations, multiplicity of analyses, results from similar studies, and other relevant evidence	16-17
Generalisability	21	Discuss the generalisability (external validity) of the study results	15
**Other information**			
Funding	22	Give the source of funding and the role of the funders for the present study and, if applicable, for the original study on which the present article is based	18

*Give information separately for cases and controls in case-control studies and, if applicable, for exposed and unexposed groups in cohort and cross-sectional studies.

Note: An Explanation and Elaboration article discusses each checklist item and gives methodological background and published examples of transparent reporting. The STROBE checklist is best used in conjunction with this article (freely available on the Web sites of PLoS Medicine at http://www.plosmedicine.org/, Annals of Internal Medicine at http://www.annals.org/, and Epidemiology at http://www.epidem.com/). Information on the STROBE Initiative is available at www.strobe-statement.org.

For peer review only

Associations of social environment, socioeconomic position and social mobility with immune response in young adults: The Jerusalem Perinatal Family Follow-up Study

List of Authors:

Gabriella M. Lawrence,[1] Yechiel Friedlander,[1] Ronit Calderon-Margalit,[1] Daniel A. Enquobahrie,[2,3] Jonathan Y. Huang,[3,4] Russell P. Tracy,[5] Orly Manor,[1] David S. Siscovick,[6] and Hagit Hochner[1]

[1]Braun School of Public Health, Hebrew University-Hadassah Medical Center, Jerusalem, Israel, [2]Cardiovascular Health Research Unit, [3]Department of Epidemiology, University of Washington, Seattle, WA,USA, [4]Institute for Health and Social Policy, McGill University, Montreal, QC, [5]Departments of Pathology and Biochemistry, University of Vermont, Burlington, VT, USA [6]New York Academy of Medicine, New York, NY, USA

Correspondence to:
Hagit Hochner, PhD
Unit of Epidemiology, Hebrew University-Hadassah Braun School of Public Health
POB 12272, Jerusalem 91120, Israel
Phone: +972-2-6777502 Fax: +972-2-6431086
Email: hagith@ekmd.huji.ac.il

Word Count: 3,233

For peer review only

For peer review only - http://bmjopen.bmj.com/site/about/guidelines.xhtml

ABSTRACT

Objectives: Immune response to cytomegalovirus (CMV) impacts adult chronic disease. This study investigates associations of childhood and adulthood social environment, socioeconomic position (SEP) and social mobility with CMV -response in young-adults.

Design: Historical prospective study design

Setting: Subcohort of all 17,003 births to residents of Jerusalem between 1974 and 1976.

Participants: Participants included 1,319 young adults born in Jerusalem with extensive archival and follow-up data, including childhood and adulthood SEP-related factors and anti-CMV IgG titer levels and seroprevalence measured at age 32.

Main exposure and outcome measures: Principal component analysis (PCA) was used to transform correlated social environment and SEP-related variables at two time points (childhood and adulthood) into two major scores reflecting household (e.g. number of siblings/children, religiosity) and socioeconomic (e.g. occupation, education) components. Based on these components, social mobility variables were created. Linear and Poisson regression models were used to investigate associations of components and mobility with anti-CMV IgG titer level and seroprevalence, adjusted for confounders.

Results: Lower levels of household and socioeconomic components in either childhood or adulthood were associated with higher anti-CMV IgG titer level and seropositivity at age 32. Compared to individuals with stable favorable components, anti-CMV IgG titer level and risk for seropositivity were higher in stable unfavorable household and socioeconomic components (household: β=3.23,$p<0.001$; RR=1.21,$p<0.001$; socioeconomic: β=2.20,$p=0.001$, RR=1.14,$p=0.01$), downward household mobility (β=4.32, $p<0.001$; RR=1.26, $p<0.001$) and upward socioeconomic mobility (β=1.37, $p=0.04$; RR=1.19, $p<0.001$). Among seropositive individuals, associations between household components and mobility with anti-CMV IgG titer level were maintained and associations between socioeconomic components and mobility with anti-CMV IgG titer level were attenuated.

For peer review only - http://bmjopen.bmj.com/site/about/guidelines.xhtml

Conclusions: Our study provides evidence that accumulating low SEP from childhood through adulthood and social mobility may compromise immune response in young adulthood

Keywords: Immune System, Epidemiology, Socioeconomic Position, Social Mobility, Cytomegalovirus, Cardiovascular

Strengths and limitations of this study

- Cytomegalovirus (CMV) antibody as a biomarker for atherosclerosis is a novel addition to studies of cardio-metabolic risk
- This is a unique population-based birth cohort with rich archival and follow-up data from birth to young adulthood
- We used an aggregate view of the social environment and SEP based on high-quality characterization in both childhood and adulthood to assess relationships at different time points in life as well as changes from childhood to adulthood with adult immune-response reflected by CMV titers
- Timing at initial seroconversion cannot be determined as only a single CMV titer measure was taken at young adulthood, also limiting precision in detection of lifetime viral load

For peer review only

INTRODUCTION

Chronic inflammation is a known risk factor for cardiovascular disease, and infection-induced inflammation may be one of the causes of endothelial dysfunction leading to atherosclerosis[1]. Specifically, immune response to cytomegalovirus (CMV), a highly transmissible beta herpesvirus endemic throughout the world [2] and a biomarker of immune response [3,4], has been implicated in the development of atherosclerosis [3,5–7] and cardiovascular-related morbidity [8,9] and mortality[5,10–12]. Until recently, non-congenital CMV was not seen as clinically relevant in immunocompetent individuals due to the lack of overt clinical symptoms. However, accumulating evidence suggests that CMV antibody titer is associated with pro-inflammatory cytokines[3,4]; more memory T-cells are devoted to suppression of latent CMV [12,13] and fewer naïve T-cells are available to respond to new infections [12–14], leading to chronic immune activation and inflammation, and subsequent cardiovascular disease. Despite the sizeable cellular response from the host, a characteristic unique to CMV, the virus adapts to the immune system efficiently. CMV, therefore, is never eliminated from the infected immunocompetent individual, in whom it causes a persistent asymptomatic infection [15,16].

Research suggests that early-life environments affect immune function over the life-course[17]. Studies show that early-life stress results in infection-related premature death, immunosuppression [18], impairment in components of cell-mediated immunity, reduction in antibody response to vaccination and elevated antibody level [17,19,20] during childhood and beyond. A life-course approach to health proposes that interaction of environment and biological factors – e.g. epigenetic programming - experienced throughout the life-course impact current and future health[21–23].

For peer review only - http://bmjopen.bmj.com/site/about/guidelines.xhtml

Additionally, research has clearly indicated that socioeconomic factors throughout the life course impact adult health[21,24–30] and particularly in Israel, religious observance, a central characteristic of the Israeli social environment, impacts adult health irrespective of socioeconomic position[31]. Associations between CMV seropositivity and antibody titer with education, income [32], household crowding [2,33] and family size [34] further indicate that social factors influence immune response. Additionally, recent studies examining the impact of social mobility and socioeconomic gradients on adult health report varied results [24,25,35,36] . While upward mobility may attenuate the harmful effects of earlier disadvantage, and downward mobility is hypothesized to have a detrimental influence on health despite advantages at earlier ages, sustained exposure to low socioeconomic position (SEP) is most commonly associated with worse health [25,36,37]. Though associations between early-life socioeconomic factors and CMV have been demonstrated, the effect of social transitions from childhood to adulthood on CMV-response has not been investigated.

This study investigates associations between SEP in childhood and adulthood and social mobility from birth to 32 years with CMV-response in young-adults of the Jerusalem Perinatal Family Follow-up Study.

METHODS

The Jerusalem Perinatal Study (JPS) population-based cohort includes a subcohort of all 17,003 births to residents of Jerusalem between 1974 and 1976 [38,39]. Data consist of demographic and socioeconomic information, maternal medical conditions during current and previous pregnancies, and offspring birth-weight, abstracted from birth certificates or maternity ward logbooks. Additional information, including socioeconomic-related factors, gestational-age,

For peer review only - http://bmjopen.bmj.com/site/about/guidelines.xhtml

smoking, pre-pregnancy and end of pregnancy weight, and gynecological history, was collected by interviewing mothers on first or second postpartum day. Detailed information on data collection is previously described [38,39].

The JPS Family Follow-Up study includes a sample of 1,400 offspring from the 1974-1976 cohort interviewed and examined between 2007-2009 (mean age=32; range: 30-35 years) [40]. Briefly, sampling frame included singletons and term (gestational age ≥36 weeks) births without congenital malformations stratified by maternal pre-pregnancy BMI and birth-weight. Blood samples at fasting were taken and offspring IgG antibodies to CMV antigen were measured in plasma using indirect enzyme immunoassay with the Diamedix Immunosimplicity Is-CMV IgG Test Kit (Diamedex Corporation, 2008) at the University of Vermont. Further Test Kit details provided in supplement 1. Anti-CMV IgG titer levels were measured for 1,319 offspring.

This study was approved by the Institutional Review Board of the Hadassah-Hebrew University Medical Center and by the University of Washington Human Subject Review Committee. All participants provided informed consent.

Study Variables

Outcome Variables

The primary outcome examined was offspring CMV-response measured through offspring antibody to CMV at age 32. Transformation (square-root) of anti-CMV IgG titer level (EU/ml) was performed due to right skewness and treated as a continuous variable. CMV seroprevalence (seropositivity ≥10 EU/ml) was examined as a secondary dichotomous outcome.

Explanatory Variables

For peer review only - http://bmjopen.bmj.com/site/about/guidelines.xhtml

For peer review only

The multi-ethnic society of Israel[41] provides an opportunity to examine SEP alongside culturally relevant social environment characteristics, including religiosity and family-size in addition to well-recognized SEP variables such as occupation and education.

SEP-related explanatory variables were examined at birth and age 32, reflecting early (i.e., pre- and peri-natal periods) and young-adult life-course periods, respectively. Variables at childhood include (1)maternal religiosity (self-identified as ultra-orthodox, orthodox, traditional, secular and collapsed into dichotomous categories: ultra-orthodox and orthodox as religious, traditional and secular as not religious); (2)paternal lay-leadership (i.e. whether father was an ordained rabbi, dichotomous); (3)father's occupation (scale:1-6; 1-low, 6-high) as described by Corcoran[26]; (4)maternal education (years, continuous); and (5)number of siblings (continuous). Variables at adulthood include (1)offspring religiosity (self-identified as ultra-orthodox, orthodox, traditional, secular and collapsed into dichotomous categories: ultra-orthodox and orthodox as religious, traditional and secular as not religious); (2)offspring occupation (scale :1-6; 1-low, 6-high); (3)offspring education (years, continuous); and (4)parity at age 32 (continuous).

Potential confounders include offspring sex (dichotomous), maternal and paternal age at childbirth (years, continuous), maternal and paternal smoking during pregnancy and offspring smoking in young-adulthood (number of cigarettes smoked per day: 0, 1-10, 11-20, 21+).

Principal Component Analysis (PCA)

Following the successful use of PCA in creating socioeconomic indices in past and current studies[42,43], and given the available array of SEP-related variables, including SEP variables

For peer review only - http://bmjopen.bmj.com/site/about/guidelines.xhtml

well known in the literature as well as specific social environment-related variables mentioned above, and the overlap and correlation between SEP-related variables (including individual-level indicators like education, occupation-based indicators and life course SEP)[44], we used PCA to reduce the number of potentially correlated variables in our cohort to a smaller number of principle components. (See Supplement 2 for correlations between SEP-related variables in the JPS Family Follow-up cohort.) PCA [45] carried out separately for variables measured at childhood and adulthood, transformed SEP-related variables into two major components: the social environment reflected by household-related characteristics (e.g. number of siblings/children, religiosity) and the socioeconomic (e.g. occupation, education) environment. The two components together explained 74% of childhood and 75% of adulthood variance. In addition to capturing the largest possible variance, these components nicely portray two elements of SEP in Israeli society – the socioeconomic element characterized by education and occupation as is often the case in many societies, and the social environment element, unique to Israeli society, in which families that identify as more religious tend to have more children[46], with the most religious families often among those with lower income level[47]. Detailed information on component construction and variables included in PCA provided in supplement 3. Higher component score indicates improvement in SEP – i.e. higher household component score indicates fewer children/decreased religiosity and higher socioeconomic component score indicates higher occupation level/years of education.

Social Mobility

For both household and socioeconomic components, childhood to adulthood social mobility variables were created. Components were divided into tertiles and dichotomous variables were

For peer review only - http://bmjopen.bmj.com/site/about/guidelines.xhtml

created for each component (lowest tertile = low; middle and high tertile = high). Four categories of childhood to adulthood transition were created separately for household and socioeconomic component mobility: stable favorable (high-high), upward mobility (low-high), downward mobility (high-low), and stable unfavorable (low-low).

Statistical Analyses

Analysis of variance model, adjusted for sex, was used to compare mean anti-CMV IgG titer level by categories of characteristics obtained at birth and age 32. Linear regression models were used to investigate associations of childhood and adulthood household and socioeconomic components as well as social mobility with anti-CMV IgG titer level measured at age 32. Multivariable models were fitted to assess associations between childhood and adulthood household and socioeconomic components, independently of each other, with anti-CMV IgG titer controlling for sex, parental ages at offspring birth and parental and offspring smoking. Multivariable models were also fitted to examine associations between individual socioeconomic and household-related variables (i.e. father's occupation, maternal education, maternal religiosity, paternal lay leadership, number of siblings, offspring occupation, offspring education, offspring religiosity and parity), independently of each other, with anti-CMV IgG titer controlling for sex, parental ages at offspring birth and parental and offspring smoking. Similar multivariable models were fitted to assess associations between household and socioeconomic mobility with anti-CMV IgG titer controlling for sex, parental ages at offspring birth and parental and offspring smoking. Poisson regression models with robust error variance were used to estimate relative risk of CMV seropositivity for each unit increase in component scores and among categories of social mobility variables. In order to verify that associations between

For peer review only - http://bmjopen.bmj.com/site/about/guidelines.xhtml

socioeconomic factors and CMV titer levels are not primarily a reflection of associations between serostatus and CMV, all analyses were carried out for the total population (both seropositive and seronegative) and additionally among only the CMV seropositive population.

All models used inverse probability weighting to account for stratified sampling. Analyses were carried out using Stata 12.0 (StataCorp, College Station, TX).

RESULTS

Parental and offspring characteristics obtained at birth and offspring characteristics at age 32, including anti-CMV IgG titer, CMV seroprevalence and social mobility are listed in Table 1. Mean anti-CMV IgG titer and seropositivity were greater among females than males. Overall, 79.7% of the study population was seropositive for CMV. For social mobility, 7.2% of the population was categorized as downwardly mobile in household component and 15.2% experienced downward socioeconomic-mobility; 7.2% and 16.9% experienced upward household and socioeconomic mobility respectively.

Table 1. Study Characteristics at Birth and Age 32[a] * (N=1,319)

**Cytomegalovirus**		
Anti-CMV IgG titer level[b]		
Females	12.0	(7.9)
Males	9.5	(7.7)
Total	10.8	(7.9)
CMV Seropositivity[b] %		
Females	84.4	
Males	75.0	
Total	79.7	
**Characteristics obtained at birth**		
Maternal Religiosity %		
Ultra-Orthodox	18.4	
Orthodox	22.2	
Traditional	35.2	
Secular	24.2	

For peer review only - http://bmjopen.bmj.com/site/about/guidelines.xhtml

Paternal Lay Leadership %		
Yes	14.0	
No	86.0	
Paternal Occupation Level %		
Low	40.0	
Middle	23.3	
High	36.7	
Maternal Years of Education (years)	11.6	(3.4)
Number of Siblings %		
≤2	30.0	
3-4	39.5	
5+	30.5	
Maternal Age at Childbirth (years)	28.1	(5.6)
Paternal Age at Childbirth (years)	32.0	(6.9)
Maternal Smoking in Pregnancy %		
No	86.7	
Yes	13.3	
Paternal Smoking in Pregnancy %		
No	56.6	
Yes	43.4	
**Characteristics obtained at age 32**		
Religiosity %		
Ultra-Orthodox	20.5	
Orthodox	20.8	
Traditional	20.6	
Secular	38.1	
Occupation Level %		
Low	10.8	
Middle	33.2	
High	56.0	
Years of Education	15.5	(3.9)
Parity %		
≤2	62.6	
3-4	22.8	
5+	14.6	
Smoking %		
No	59.4	
Yes	40.6	
**Social Mobility**[d]		
Household[c] Mobility %		
Stable & Favorable	58.8	
Upward Mobility	7.1	
Downward Mobility	7.2	
Stable & Unfavorable	26.9	

For peer review only - http://bmjopen.bmj.com/site/about/guidelines.xhtml

For peer review only

Socioeconomic[c] Mobility %	
Stable & Favorable	49.5
Upward Mobility	16.9
Downward Mobility	15.2
Stable & Unfavorable	18.4

CMV = cytomegalovirus
[a]Values are expressed as mean (Standard Deviation) or percent
[b]obtained at age 32; square root transformed EU/ml; Seroprevalence = IgG titer level ≥10 EU/ml
[c]Household and Socioeconomic components created using principal component analysis detailed in methods; higher component score indicates improvement in household or socioeconomic factors
[d]Social mobility variables represent transition from childhood to adulthood using dichotomized household and socioeconomic components

Table 2 presents mean anti-CMV IgG titer level by categories of characteristics obtained at birth and age 32 as well as social mobility among the total population. Among the total population, mean anti-CMV IgG was highest among offspring born to ultra-orthodox mothers and fathers holding religious leadership positions, as well as offspring born to mothers with <9 years of education and those with ≥5 siblings. Similarly, ultra-orthodox offspring at age 32, those with ≤12 years of education and with more than 5 children had higher mean anti-CMV IgG titer level. Among CMV seropositive individuals, a similar pattern of mean anti-CMV IgG titer level by categories of characteristics obtained at birth and age 32 was found (supplement 4).

Table 2. Mean[a] Anti-CMV IgG Titer Level[b] by Categories of Characteristics Obtained at Birth and Age 32 (N=1,319)

	Mean	(SE)	P-value[e]
**Characteristics obtained at birth**			
Maternal Religiosity			<.0001
Ultra-Orthodox	12.9	(0.52)	
Orthodox	11.4	(0.47)	
Traditional	10.5	(0.37)	
Secular	8.5	(0.43)	
Paternal Lay-Leadership			<.0001
Yes	13.5	(0.23)	

For peer review only - http://bmjopen.bmj.com/site/about/guidelines.xhtml

No	10.3	(0.58)	
Paternal Occupation Level			0.8
Low	10.4	(0.34)	
Middle	10.6	(0.44)	
High	10.6	(0.35)	
Maternal Education			0.003
<9 years	11.9	(0.47)	
9-12 years	10.8	(0.33)	
>12 years	9.9	(0.36)	
Number of Siblings			<.0001
≤2	9.4	(0.38)	
3-4	10.3	(0.34)	
5+	12.8	(0.40)	
**Characteristics obtained at age 32**			
Religiosity			<0.0001
Ultra-Orthodox	12.6	(0.48)	
Orthodox	12.2	(0.48)	
Traditional	11.0	(0.48)	
Secular	9.1	(0.34)	
Occupation Level			0.01
Low	11.4	(0.68)	
Middle	11.6	(0.39)	
High	10.2	(0.29)	
Years of Education			0.001
≤ 12	11.9	(0.42)	
>12	10.3	(0.25)	
Parity			<0.0001
≤2	9.6	(0.27)	
3-4	12.5	(0.45)	
5+	13.1	(0.57)	

CMV = cytomegalovirus; SE = standard error
[a]Sex-adjusted
[b]square root transformed EU/ml
[c]Obtained through ANOVA

In order to model associations and adjust for additional covariates (i.e. offspring sex, parental ages at birth and parental and offspring smoking), we investigated associations between household and socioeconomic components with anti-CMV IgG titer using linear regression models and with CMV seroprevalence using Poisson regression models presented in Table 3.

For peer review only

For peer review only - http://bmjopen.bmj.com/site/about/guidelines.xhtml

Table 3. Regression Coefficients, Relative Risks and 95% CI of CMV in Relation to Household and Socioeconomic Components[c] in Childhood and Adulthood

	Continuous CMV[a]						Seroprevalence[b]					
	Model 1[d]			Model 2[d]			Model 1[d]			Model 2[d]		
**Component[c]**	**Coef.[d]**	***P***	**95% CI**	**Coef.[d]**	***P***	**95% CI**	**RR[d]**	***P***	**95% CI**	**RR[d]**	***P***	**95% CI**
Household												
Childhood	-0.78	<0.001	-1.11,-0.45	-0.07	0.76	-0.53,0.39	0.96	0.001	0.94,0.98	1.00	0.86	0.97,1.04
Adulthood	-1.25	<0.001	-1.61,-0.90	-1.02	<0.001	-1.55,-0.49	0.94	<0.001	0.91,0.97	0.95	0.02	0.91,0.99
Socioeconomic												
Childhood	-1.04	<0.001	-1.43,-0.65	-0.74	0.001	-1.17,-0.31	0.91	<0.001	0.88,0.95	0.94	0.001	0.90,0.98
Adulthood	-0.71	<0.001	-1.09,-0.33	-0.39	0.08	-0.82,0.04	0.93	<0.001	0.90,0.97	0.95	0.02	0.92,0.99

Coef. = coefficient; CI = confidence interval; CMV = cytomegalovirus; RR = relative risk

[a]continuous square root transformed EU/ml

[b]Anti-CMV IgG titer level ≥10 EU/ml

[c]Created using principal component analysis detailed in methods

[d]Model 1 includes each component separately. Model 2 includes all four components together in model. All models adjusted for sex, maternal and paternal age at offspring birth and maternal, paternal and offspring smoking

[d]Coefficient = change in anti-CMV IgG titer level per increase in one unit of component (distribution characteristics in supplement 3); RR = change in risk estimate of CMV seropositivity per increase in one unit of component – calculated by Poisson regression with robust error variance

Lower levels of household and socioeconomic components in childhood and adulthood were associated with higher anti-CMV IgG titer level at age 32 (Table 3, continuous CMV Model 1) and increased CMV seropositivity (Table 3, seroprevalence Model 1). When childhood and adulthood components were included together in one model (Table 3, continuous CMV Model 2), only adulthood household and childhood socioeconomic components were associated with anti-CMV IgG titer (β= -1.02, p<0.001 and β= -0.74,

For peer review only - http://bmjopen.bmj.com/site/about/guidelines.xhtml

p=0.001, respectively). Among seropositive individuals, associations between household components and anti-CMV IgG titer level were maintained and associations between socioeconomic components and anti-CMV IgG titer level were attenuated (supplement 5). Models looking at associations between individual socioeconomic and household-related variables with anti-CMV IgG titer level found similar results to PCA-based models (supplement 6). We also investigated associations between household and socioeconomic component-mobility and anti-CMV IgG titer, adjusted for sex, parental ages at offspring birth and parental and offspring smoking (Figure 1). Compared to individuals with stable favorable household components, mean anti-CMV IgG levels were higher in those who maintained unfavorable household components from childhood to adulthood (β=3.23, p<0.001) and those who transitioned from a favorable to unfavorable position (i.e. downward mobility) (β=4.32, p<0.001). Those who maintained a stable unfavorable socioeconomic component position had increased anti-CMV titer level compared to those in the stable favorable position (β=2.20, p=0.001) as did those in the upward mobility category (β=1.37, p=0.04). A similar pattern of associations was seen when investigating associations between mobility and CMV seroprevalence (Figure 1). Among CMV seropositive individuals, similar associations were found between household component mobility and anti-CMV IgG titer and associations between socioeconomic component mobility and anti-CMV IgG titer level were primarily attenuated (supplement 7).

DISCUSSION

This study demonstrated that lower levels of household and socioeconomic components in childhood and adulthood, reflecting lower SEP, were associated with higher anti-CMV IgG titer

For peer review only - http://bmjopen.bmj.com/site/about/guidelines.xhtml

level at age 32. Additionally, maintaining a stable unfavorable position from childhood to adulthood was associated with higher anti-CMV IgG titer level compared to the stable favorable position. Our study also provides some evidence that social mobility transitions from childhood to adulthood (i.e. upward and downward mobility) may be associated with CMV-response.

Life-Course Models

Life-course models explaining the impact of SEP on adult health proposed in the literature include the critical period model, emphasizing timing of exposure[48,49], the social mobility model, focusing on transitions across the life-course[48,49], and the accumulation model, emphasizing duration of exposures[48,49]. As per the critical period model, our findings that lower childhood and adulthood household and socioeconomic components are associated with higher anti-CMV IgG titer level in early adulthood are in accordance with other studies[17,27]. Our study also adds to the social mobility model, though unlike others[24,28,35], we found that upward socioeconomic mobility was associated with higher CMV titer and higher risk for CMV seropositivity. Possibly, as others, including JPS analyses, have found[29], mechanisms similar to those in Gluckman's "Mismatch hypothesis" are at play – in which early environment induces long-term cardiovascular effects if there is a "mismatch" (including improvement) between early developmental environment and subsequent environment in later life. Household downward mobility, as in other cohort-based research[25], was associated with increased CMV IgG titer levels and increased risk for CMV seropositivity. Our study is the first to investigate social mobility associations with young-adult CMV-response.

For peer review only - http://bmjopen.bmj.com/site/about/guidelines.xhtml

Research has indicated that sustained exposure to low SEP has been most commonly associated with worse health[25,28,35–37,48]. Our study supports the detrimental impact of accumulation of lower SEP over the life-course, as we found increased anti-CMV IgG titer and risk for CMV seropositivity among those in both the household and socioeconomic stable unfavorable group as compared to stable favorable.

Multidimensionality of Exposure

The associations of social mobility with young-adult CMV-response differ somewhat in household and socioeconomic components. While household downward mobility was associated with increased anti-CMV IgG titer level, upward socioeconomic mobility seemingly had a harmful impact on immune response at age 32. These findings highlight the multidimensionality of socioeconomic measurement and the varying contribution of different socioeconomic dimensions through the life-course[30]. The PCA used in our analyses may provide a more aggregate characterization of the socioeconomic experience/environment, while demonstrating that various socioeconomic factors may indeed represent different domains. To further highlight this point, household upward or downward mobility comprised only 14.3% of the study population while socioeconomic mobility was seen among 31.1%, possibly indicating that the household component is more stable than the socioeconomic component. Possibly, people tend to maintain a level of religiosity similar to that with which they were raised[50] whereas socioeconomic transitions from parents to offspring vary depending on culture, environment, psychological elements and geographic residence[51].

Epigenetic Mechanism

For peer review only - http://bmjopen.bmj.com/site/about/guidelines.xhtml

Previous studies found that participants originating from low and high SEP have different patterns of methylation[52]. Additionally, associations have been found between methylation and chronic inflammation and a previous analysis of JPS demonstrated an *HSD11B2*–cardiometabolic risk association[23]. Epigenetic changes in response to environment may play an important role in the development of adult diseases[53]. Further studies that investigate an epigenetic role in the SEP–CMV associations may provide evidence for mechanisms that explain these associations.

Study Strengths and Limitations

One of the major strengths of this study is the novel use of CMV antibody, emerging as a potential biomarker for atherosclerosis and cardiometabolic risk, as well as the use of continuous CMV antibody in addition to CMV seropositivity as a study outcome. Few studies have examined CMV antibody titer on a continuous scale, despite potential epidemiological significance of such an outcome. Additionally, very few studies have had the ability to examine the impact of SEP and social mobility on immune-response in young adulthood. The combination of high-quality detailed records of perinatal maternal and offspring characteristics and long-term follow-up data at age 32, improved the characterization of the socioeconomic environment surrounding pregnancy, childhood and adulthood, and allowed analyses to examine changes from childhood to adulthood. Estimating different components of the socioeconomic environment and trajectory by PCA was another novel approach, enabling us to capture common variance and providing an aggregate view of the socioeconomic experience/environment, rather than limiting analyses to individual SEP proxies.

For peer review only - http://bmjopen.bmj.com/site/about/guidelines.xhtml

Our study has several limitations. First, it includes only a sample of offspring from the original 1974-1976 JPS cohort invited to participate in the follow-up study. However, using a stratified sampling approach and over-sampling ends of the distribution ensured that offspring with a range of fetal and early-life characteristics were included in our study. Second, examining CMV titer on a continuous scale using qualitative ELISA assay limits clinical interpretation of findings. However, our findings using CMV seropositivity, a clinically relevant diagnosis, support the associations found with CMV antibody titer, and substantiate the use of continuous CMV antibody in future epidemiological studies. Importantly, this specific assay was successfully used in other cohorts, such as the Multi-Ethnic Study of Atherosclerosis[12]. It is important to note that while the immune response to CMV has an extensive impact on the immune system, using CMV antibody titer as a proxy measure of the general cellular immune condition may not be ideal, particularly given the unique cellular response to CMV from the host. More modern approaches, using multiple technological platforms, where the multiple responding cells and cytokines in the blood can be measured[54], may give a more in-depth understanding of the overall immune response. Additionally, data consists of a single CMV titer measure for each participant. Verification of titer level from additional tests or other sources was not available, and timing/age at initial seroconversion is unknown. However, research has indicated that CMV antibody titer remains relatively stable over time[55,56], and seroprevalence rates in our population fall in the range of prevalence rates in similar populations[2,15,16,57]. Furthermore, the use of PCA may mask distinctions in relationships between individual SEP variables and CMV. However, additional analyses revealed that associations with the individual SEP variables are in line with what has been previously reported in the literature[32] and similar to PCA-

For peer review only - http://bmjopen.bmj.com/site/about/guidelines.xhtml

based results, found negative associations between education and anti-CMV IgG titer level, particularly in childhood and positive associations between religiosity and family size with anti-CMV IgG titer particularly in adulthood. PCA was used to reduce the number of potentially correlated variables in our cohort to a smaller number of principle components that would produce a more coherent picture of the SEP-CMV relationship. While relationships between SEP and CMV have been reported in studies in other populations, the differing associations found in our study between socioeconomic vs. household components, may be particular to unique elements in Israeli society.

Conclusions

Our study extends accumulating evidence of relationships between socioeconomic factors in childhood and adulthood with adult immune response. Additionally, our study provides evidence that accumulating low SEP from childhood through adulthood and social mobility may compromise immune response in young adulthood. Further studies should explore mechanisms underlying the relationships between childhood environments and social mobility with immunity in adulthood. A better understanding of these relationships may lead to new approaches to counteract effects of low-SEP environments through the life-course and improve immune response in high-risk young-adults.

For peer review only - http://bmjopen.bmj.com/site/about/guidelines.xhtml

REFERENCES

1 Ross R. Inflammation or Atherogenesis. *N Engl J Med* 1999;**340**:115–26. doi:10.1056/NEJM199901143400207

2 Bate SL, Dollard SC, Cannon MJ. Cytomegalovirus seroprevalence in the United States: the national health and nutrition examination surveys, 1988-2004. *Clin Infect Dis* 2010;**50**:1439–47. doi:10.1086/652438

3 Nazmi A, Diez-Roux A V, Jenny NS, *et al.* The influence of persistent pathogens on circulating levels of inflammatory markers: a cross-sectional analysis from the Multi-Ethnic Study of Atherosclerosis. *BMC Public Health* 2010;**10**:706. doi:10.1186/1471-2458-10-706

4 Marashi SM, Raeiszadeh M, Enright V, *et al.* Influence of cytomegalovirus infection on immune cell phenotypes in patients with common variable immunodeficiency. *J Allergy Clin Immunol* 2012;**129**:1349–1356.e3. doi:10.1016/j.jaci.2012.02.011

5 Simanek AM, Dowd JB, Pawelec G, *et al.* Seropositivity to cytomegalovirus, inflammation, all-cause and cardiovascular disease-related mortality in the United States. *PLoS One* 2011;**6**:e16103. doi:10.1371/journal.pone.0016103

6 Zhu J, Quyyumi A a, Norman JE, *et al.* Cytomegalovirus in the pathogenesis of atherosclerosis. *J Am Coll Cardiol* 1999;**34**:1738–43. doi:10.1016/S0735-1097(99)00410-6

7 Blum a., Peleg a., Weinberg M. Anti-cytomegalovirus (CMV) IgG antibody titer in patients with risk factors to atherosclerosis. *Clin Exp Med* 2003;**3**:157–60. doi:10.1007/s10238-003-0019-7

8 Ji Y-N, An L, Zhan P, *et al.* Cytomegalovirus infection and coronary heart disease risk: a meta-analysis. *Mol Biol Rep* 2012;**39**:6537–46. doi:10.1007/s11033-012-1482-6

9 Zhang M, Yang Y, Yang X, *et al.* Human cytomegalovirus infection is a novel etiology for essential hypertension. *Med Hypotheses* 2011;**76**:682–4. doi:10.1016/j.mehy.2011.01.032

10 Roberts ET, Haan MN, Dowd JB, *et al.* Cytomegalovirus antibody levels, inflammation, and mortality among elderly Latinos over 9 years of follow-up. *Am J Epidemiol* 2010;**172**:363–71. doi:10.1093/aje/kwq177

11 Strandberg TE, Pitkala KH, Tilvis RS. Cytomegalovirus antibody level and mortality among community-dwelling older adults with stable cardiovascular disease. *JAMA* 2009;**301**:380–2. doi:10.1001/jama.2009.4

12 Tracy RP, Doyle MF, Olson NC, *et al.* T-helper type 1 bias in healthy people is associated with cytomegalovirus serology and atherosclerosis: the Multi-Ethnic Study of Atherosclerosis. *J Am Heart Assoc* 2013;**2**:e000117. doi:10.1161/JAHA.113.000117

13 Olson NC, Doyle MF, Jenny NS, *et al.* Decreased naive and increased memory CD4(+) T cells are associated with subclinical atherosclerosis: the multi-ethnic study of atherosclerosis. *PLoS One* 2013;**8**:e71498. doi:10.1371/journal.pone.0071498

14 Zhu J. Discordant cellular and humoral immune responses to cytomegalovirus infection in

For peer review only - http://bmjopen.bmj.com/site/about/guidelines.xhtml

healthy blood donors: existence of a Th1-type dominant response. *Int Immunol* 2001;**13**:785–90. doi:10.1093/intimm/13.6.785

15 La Rosa C, Diamond DJ. The immune response to human CMV. *Future Virol* 2012;**7**:279–93. doi:10.2217/fvl.12.8

16 Levine H, Balicer RD, Rozhavski V, *et al.* Seroepidemiology of Epstein-Barr virus and cytomegalovirus among Israeli male young adults. *Ann Epidemiol* 2012;**22**:783–8. doi:10.1016/j.annepidem.2012.06.099

17 McDade TW. Life history, maintenance, and the early origins of immune function. *Am J Hum Biol* 2005;**17**:81–94. doi:10.1002/ajhb.20095

18 Moore SE, Cole TJ, Collinson AC, *et al.* Prenatal or early postnatal events predict infectious deaths in young adulthood in rural Africa. *Int J Epidemiol* 1999;**28**:1088–95.

19 Shirtcliff EA, Coe CL, Pollak SD. Early childhood stress is associated with elevated antibody levels to herpes simplex virus type 1. *Proc Natl Acad Sci U S A* 2009;**106**:2963–7. doi:10.1073/pnas.0806660106

20 McDade TW, Stallings JF, Angold A, *et al.* Epstein-Barr virus antibodies in whole blood spots: a minimally invasive method for assessing an aspect of cell-mediated immunity. *Psychosom Med* 2000;**62**:560–7.

21 Power C, Atherton K, Strachan DP, *et al.* Life-course influences on health in British adults: effects of socio-economic position in childhood and adulthood. *Int J Epidemiol* 2007;**36**:532–9. doi:10.1093/ije/dyl310

22 Borghol N, Suderman M, McArdle W, *et al.* Associations with early-life socio-economic position in adult DNA methylation. *Int J Epidemiol* 2012;**41**:62–74. doi:10.1093/ije/dyr147

23 Huang JY, Gavin AR, Richardson TS, *et al.* Accounting for life course exposures in epigenetic biomarker association studies: Early life socioeconomic position, candidate gene DNA methylation, and adult cardiometabolic risk. *Am J Epidemiol* 2016;**in-press**.

24 Aitsi-Selmi A, Batty GD, Barbieri MA, *et al.* Childhood socioeconomic position, adult socioeconomic position and social mobility in relation to markers of adiposity in early adulthood: evidence of differential effects by gender in the 1978/79 Ribeirao Preto cohort study. *Int J Obes (Lond)* 2013;**37**:439–47. doi:10.1038/ijo.2012.64

25 Smith BT, Lynch JW, Fox CS, *et al.* Life-course socioeconomic position and type 2 diabetes mellitus: The Framingham Offspring Study. *Am J Epidemiol* 2011;**173**:438–47. doi:10.1093/aje/kwq379

26 Corcoran C, Perrin M, Harlap S, *et al.* Effect of socioeconomic status and parents' education at birth on risk of schizophrenia in offspring. Soc. Psychiatry Psychiatr. Epidemiol. 2009;**44**:265–71. doi:10.1007/s00127-008-0439-5

27 Demakakos P, Marmot M, Steptoe A. Socioeconomic position and the incidence of type 2 diabetes: The ELSA study. *Eur J Epidemiol* 2012;**27**:367–78. doi:10.1007/s10654-012-9688-4

For peer review only - http://bmjopen.bmj.com/site/about/guidelines.xhtml

28 Albrecht SS, Gordon-Larsen P. Socioeconomic gradients in body mass index (BMI) in US immigrants during the transition to adulthood: examining the roles of parental education and intergenerational educational mobility. *J Epidemiol Community Health* 2014;**68**:842–8. doi:10.1136/jech-2014-203814

29 Savitsky B, Manor O, Friedlander Y, *et al.* Associations of socioeconomic position in childhood and young adulthood with cardiometabolic risk factors: the Jerusalem Perinatal Family Follow-Up Study. *J Epidemiol Community Health* 2017;**71**:43–51. doi:10.1136/jech-2014-204323

30 Cutler DM, Lleras-Muney A VT. Socioeconomic status and health: Dimensions and mechanisms. *Oxford Handb Heal Econon* 2011;:124–163.

31 Kark JD, Shemi G, Friedlander Y, *et al.* Does Religious Observance Promote Health ? Mortality in Secular vs Religious Kibbutzim in Israel. 1995.

32 Dowd JB, Aiello AE. Socioeconomic differentials in immune response. *Epidemiology* 2009;**20**:902–8. doi:10.1097/EDE.0b013e3181bb5302

33 Jansen MAE, van den Heuvel D, Bouthoorn SH, *et al.* Determinants of Ethnic Differences in Cytomegalovirus, Epstein-Barr Virus, and Herpes Simplex Virus Type 1 Seroprevalence in Childhood. *J Pediatr* 2016;**170**:126–34. doi:10.1016/j.jpeds.2015.11.014

34 Shigemi D, Yamaguchi S, Otsuka T, *et al.* Seroprevalence of cytomegalovirus IgG antibodies among pregnant women in Japan from 2009-2014. *Am J Infect Control* 2015;**43**:1218–21. doi:10.1016/j.ajic.2015.06.026

35 Johnson-Lawrence V, Kaplan G, Galea S. Socioeconomic mobility in adulthood and cardiovascular disease mortality. *Ann Epidemiol* 2013;**23**:167–71. doi:10.1016/j.annepidem.2013.02.004

36 Heraclides A, Brunner E. Social mobility and social accumulation across the life course in relation to adult overweight and obesity: the Whitehall II study. *J Epidemiol Community Health* 2010;**64**:714–9. doi:10.1136/jech.2009.087692

37 Halonen JI, Stenholm S, Pentti J, *et al.* Childhood Psychosocial Adversity and Adult Neighborhood Disadvantage as Predictors of Cardiovascular Disease: A Cohort Study. *Circulation* 2015;**132**:371–9. doi:10.1161/CIRCULATIONAHA.115.015392

38 Davies AM, Prywes R, Tzur B, *et al.* The Jerusalem perinatal study. 1. Design and organization of a continuing, community-based, record-linked survey. *Isr J Med Sci* 1969;**5**:1095–106.

39 Harlap S, Davies A, Deutsch L, *et al.* The Jerusalem Perinatal Study cohort, 1964-2005: methods and a review of the main results. *Paediatr Perinat Epidemiol* 2007;**21**:256–73. doi:10.1111/j.1365-3016.2007.00799.x.The

40 Lawrence GM, Siscovick DS, Calderon-Margalit R, *et al.* Cohort Profile: The Jerusalem Perinatal Family Follow-Up Study. *Int J Epidemiol* 2016;**45**:343–52. doi:10.1093/ije/dyv120

41 Haj M Al. *Immigration and Ethnic Formation in a Deeply Divided Society: The Case of the*

For peer review only - http://bmjopen.bmj.com/site/about/guidelines.xhtml

1990s Immigrants from the Former Soviet Union in Israel. BRILL 2004.

42 Vyas S, Kumaranayake L. Constructing socio-economic status indices: How to use principal components analysis. *Health Policy Plan* 2006;**21**:459–68. doi:10.1093/heapol/czl029

43 Becher H, Palm F, Aigner A, *et al.* Socioeconomic conditions in childhood, adolescence, and adulthood and the risk of ischemic stroke. *Stroke* 2016;**47**:173–9. doi:10.1161/STROKEAHA.115.011523

44 Galobardes B, Lynch J, Smith GD. Measuring socioeconomic position in health research. *Br Med Bull* 2007;**81–82**:21–37. doi:10.1093/bmb/ldm001

45 Stevens J. *Applied multivariate statistics for the social sciences*. L. Erlbaum Associates Inc. 1986.

46 Bystrov E. Religiosity, nationalism and fertility among Jews in Israel revisited. *Acta Sociol* 2016;**59**:171–86. doi:10.1177/0001699316628615

47 Bleikh H, Berrebi C, Brand G, *et al.* Poverty and Inequality in Israel: Trends and Decompositions.

48 Pollitt RA, Rose KM, Kaufman JS. Evaluating the evidence for models of life course socioeconomic factors and cardiovascular outcomes: a systematic review. *BMC Public Health* 2005;**5**:7. doi:10.1186/1471-2458-5-7

49 Kuh D, Ben-Shlomo Y, Lynch J, *et al.* Life course epidemiology. *J Epidemiol Community Health* 2003;**57**:778–83.

50 Ashton MC. *Individual Differences and Personality*. Academic Press 2013.

51 *From Parents to Children: The Intergenerational Transmission of Advantage*. Russell Sage Foundation 2012.

52 Lam LL, Emberly E, Fraser HB, *et al.* Factors underlying variable DNA methylation in a human community cohort. *Proc Natl Acad Sci U S A* 2012;**109 Suppl**:17253–60. doi:10.1073/pnas.1121249109

53 Gilbert ER, Liu D. Epigenetics: the missing link to understanding β-cell dysfunction in the pathogenesis of type 2 diabetes. *Epigenetics* 2012;**7**:841–52. doi:10.4161/epi.21238

54 Furman D, Davis MM. New approaches to understanding the immune response to vaccination and infection. *Vaccine* 2015;**33**:5271–81. doi:10.1016/j.vaccine.2015.06.117

55 Zanghellini F, Boppana SB, Emery VC, *et al.* Asymptomatic primary cytomegalovirus infection: virologic and immunologic features. *J Infect Dis* 1999;**180**:702–7. doi:10.1086/314939

56 Miller MS, Gardner TJ, Krammer F, *et al.* Neutralizing antibodies against previously encountered influenza virus strains increase over time: a longitudinal analysis. *Sci Transl Med* 2013;**5**:198ra107. doi:10.1126/scitranslmed.3006637

57 Cannon MJ, Schmid DS, Hyde TB. Review of cytomegalovirus seroprevalence and demographic characteristics associated with infection. *Rev Med Virol* 2010;**20**:202–13.

For peer review only - http://bmjopen.bmj.com/site/about/guidelines.xhtml

doi:10.1002/rmv

For peer review only

For peer review only - http://bmjopen.bmj.com/site/about/guidelines.xhtml

Contributors: DSS, HH and YF conceived and designed the study. HH designed data collection tools and developed protocols.GML designed the analysis plan, conducted the analysis and wrote the manuscript with guidance from YF and HH. DSS and RPT advised on the clinical context of the study and analyses.OM, RC-M, DAE and JYH advised on data analysis and interpretation of findings. All authors contributed intellectually to the manuscript, reviewed and edited the manuscript and approved the final version.

Funding: This work was financially supported by the National Institutes of Health [grant numbers R01HL088884 and K01HL103174] and the Israeli Science Foundation [grant numbers 1252/07 and 552/12].

Disclaimer: The founders had no role in the design, analysis or writing of this article

Competing interests: None declared

Informed Consent: Informed consent was obtained from all individual participants included in the study.
Ethical Approval: This study was approved by the Institutional Review Board of the Hadassah-Hebrew University Medical Center and by the University of Washington Human Subject Review Committee.

Data sharing statement: Data are available on request. Email Dr. Hagit Hochner: hagith@ekmd.huji.ac.il

Figure Legend
Fig. Predicted mean anti-CMV IgG titer differences and CMV seropositivity risk by household and socioeconomic mobility. Predicted mean differences calculated via β coefficients from linear regression models adjusted for sex, maternal and paternal age at offspring birth and maternal, paternal and offspring smoking. Relative risk estimation by Poisson regression with robust error variance models adjusted for sex, maternal and paternal age at offspring birth and maternal, paternal and offspring smoking.

For peer review only - http://bmjopen.bmj.com/site/about/guidelines.xhtml

For peer review only

Supplement 1. Diamedix Immunosimplicity Is-CMV IgG Test Kit

(Diamedex Corporation, 2008)

The Test Kit is intended for the qualitative and semi-quantitative detection of antibodies to CMV antigen. Compared to currently used testing methods in three different United States-based laboratories, the sensitivity and specificity of the Diamedix Kit ranged from 99.3%-100.0% and 87.7%-100.0% respectively, and overall agreement ranged from 97.0%-99.0%. Seroprevalence results detected using this kit were in agreement with prevalence rates reported in United States blood donor populations.

Diamedix. CMV IgG: Enzyme Immunoassay Test Kit. 2008;3–6.

For peer review only

Supplement 2. Correlations between SEP-related variables in the JPS Family Follow-up cohort

	Father's occupation[a]	Maternal education[b]	Maternal religiosity[c]	Paternal lay-leadership[c]	Number of siblings[b]	Offspring occupation[a]	Offspring education[b]	Offspring religiosity[c]	Offspring parity[b]
Father's occupation[a]	1.00								
Maternal education[b]	0.52	1.00							
Maternal religiosity[c]	0.23	0.07	1.00						
Paternal lay-leadership[c]	0.32	0.10	0.49	1.00					
Number of siblings[b]	0.10	-0.14	0.54	0.60	1.00				
Offspring occupation[a]	0.22	0.29	0.00	0.02	-0.10	1.00			
Offspring education[b]	0.30	0.32	0.19	0.22	0.08	0.38	1.00		
Offspring religiosity[c]	0.19	0.06	0.72	0.47	0.53	0.00	0.18	1.00	
Offspring parity[b]	0.14	-0.04	0.50	0.49	0.54	-0.05	0.06	0.57	1.00

[a]In 6 categories; Spearman's rank correlation coefficient
[b]Continuous; Pearson correlation coefficient
[c]Dichotomous; Pearson correlation coefficient

For peer review only

Supplement 3. Principal Component Analysis Loadings

Childhood			Adulthood		
	Component Loadings			Component Loadings	
Variables Included	Household	Socioeconomic	Variables Included	Household	Socioeconomic
Paternal Occupation	0.112	0.656	Offspring Occupation	-0.111	0.719
Maternal Education	-0.132	0.723	Offspring Education	0.115	0.690
Maternal Religiosity	0.538	0.048	Offspring Religiosity	0.695	0.056
Paternal Lay Leadership	0.550	0.119			
Number of Siblings	0.616	-0.180	Offspring Parity	0.701	-0.065
Variance Explained	74%		Variance Explained	75%	

Principal Component Score Distribution

	Mean	SD	Minimum	Maximum
**Household Components**				
Childhood	-0.003	1.49	-4.63	1.86
Adulthood	-0.008	1.27	-4.05	2.08
**Socioeconomic Components**				
Childhood	0.04	1.21	-4.14	3.52
Adulthood	0.06	1.15	-3.66	3.02

For peer review only

Supplement 4. Mean[a] Anti-CMV IgG Titer Level[b] by Categories of Characteristics Obtained at Birth and Age 32 Among CMV seropositive individuals (N=1,051)

	Mean	(SE)	P-value[e]
**Characteristics obtained at birth**			
Maternal Religiosity			0.007
Ultra-Orthodox	14.34	(6.29)	
Orthodox	13.69	(7.09)	
Traditional	12.78	(6.34)	
Secular	12.37	(5.90)	
Paternal Lay-Leadership			0.06
Yes	14.12	(6.06)	
No	13.09	(6.52)	
Paternal Occupation Level			0.69
Low	13.11	(6.69)	
Middle	13.14	(6.53)	
High	13.48	(6.14)	
Maternal Education			0.26
<9 years	13.82	(7.02)	
9-12 years	13.07	(6.35)	
>12 years	13.00	(6.08)	
Number of Siblings			0.003
≤2	13.02	(6.48)	
3-4	12.92	(6.46)	
5+	13.72	(6.30)	
**Characteristics obtained at age 32**			
Religiosity			0.03
Ultra-Orthodox	13.70	(6.04)	
Orthodox	14.00	(7.08)	
Traditional	13.12	(6.45)	
Secular	12.52	(6.20)	
Occupation Level			0.06
Low	12.56	(6.12)	
Middle	13.90	(7.02)	
High	13.01	(6.12)	
Years of Education			0.32
≤ 12	13.53	(6.63)	
>12	13.10	(6.33)	
Parity			0.008
≤2	12.75	(6.44)	
3-4	13.73	(6.70)	
5+	14.07	(5.69)	

CMV = cytomegalovirus; SE = standard error

[a]Sex-adjusted

[b]square root transformed EU/ml

[c]Obtained through ANOVA

For peer review only

Supplement 5. Regression Coefficients and 95% CI of CMV in Relation to Household and Socioeconomic Components[b] in Childhood and Adulthood Among CMV Seropositive Population

	Continuous CMV[a]					
	Model 1[c]			Model 2[c]		
**Component[b]**	**Coef.[d]**	***P***	**95% CI**	**Coef.[d]**	***P***	**95% CI**
Household						
Childhood	-0.41	0.008	-0.71, -0.11	-0.03	0.91	-0.44, 0.39
Adulthood	-0.74	<0.001	-1.07, -0.42	-0.67	0.007	-1.15, -0.18
Socioeconomic						
Childhood	-0.23	0.24	-0.61, 0.15	-0.21	0.31	-0.63, 0.20
Adulthood	-0.01	0.94	-0.35, 0.38	-0.17	0.42	-0.25, 0.59

Coef. = coefficient; CI = confidence interval; CMV = cytomegalovirus
[a]Continuous square root transformed EU/ml
[b]Created using principal component analysis detailed in methods
[c]Model 1 includes each component separately. Model 2 includes all four components together in model. All models adjusted for sex, maternal and paternal age at offspring birth and maternal, paternal and offspring smoking
[d]Coefficient = change in anti-CMV IgG titer level per increase in one unit of component (distribution characteristics in supplement 3)

For peer review only

Supplement 6. Associations between social environment and socioeconomic factors with CMV antibody in total and CMV seropositive population

	Among Total Population						Among CMV Seropositive Only					
	Univariate Model[a]			Multivariate Model[b]			Univariate Model[a]			Multivariate Model[b]		
	B	P-value	95% CI	β	P-value	95% CI	B	P-value	95% CI	β	P-value	95% CI
**Characteristics at birth**												
Maternal education (yrs)[d]	-0.32	<0.001	-0.45, -0.19	-0.35	<0.001	-0.52, -0.19	-0.11	0.08	-0.23, 0.01	-0.22	0.007	-0.37, -0.06
Maternal religiosity[c]	2.28	<0.001	1.39, 3.17	-0.47	0.51	-1.89, 0.94	1.17	0.005	0.35, 1.99	-0.30	0.65	-1.59, 0.99
Paternal lay leadership[c]	2.40	<0.001	1.09, 3.71	-0.82	0.39	-2.70, 1.05	0.88	0.13	-0.26, 2.04	-0.47	0.59	-2.18, 1.24
Paternal SEP[d]	-0.09	0.46	-0.34, 0.15	0.16	0.31	-0.15, 0.48	0.12	0.31	-0.11, 0.35	0.22	0.15	-0.08, 0.54
Family size[d]	0.51	<0.001	0.34, 0.67	0.18	0.16	-0.07, 0.44	0.16	0.04	0.007, 0.30	0.01	0.91	-0.22, 0.25
**Characteristics at age 32**												
Education (yrs)[d]	-0.15	0.01	-0.27, -0.04	-0.07	0.36	-0.21, 0.08	0.01	0.81	-0.10, 0.12	0.10	0.18	-0.04, 0.24
Religiosity[c]	2.74	<0.001	1.89, 3.60	2.24	0.002	0.82, 3.66	1.55	<0.001	0.77, 2.34	1.88	0.004	0.60, 3.17
Current Family size[d]	0.79	<0.001	0.58, 1.00	0.22	0.15	-0.08, 0.52	0.28	0.005	0.08, 0.48	-0.13	0.37	-0.42, 0.16
Current SEP[d]	-0.01	0.92	-0.31, 0.28	0.09	0.58	-0.24, 0.43	0.20	0.15	-0.07, 0.48	0.08	0.64	-0.24, 0.39

[a]Univariate model includes each variable alone in model
[b]Multivariate model includes all variables together in one model + offspring sex, maternal and paternal age at childbirth, maternal and paternal smoking during pregnancy and offspring smoking in young-adulthood
[c]Treated as dichotomous variables
[d]Treated as continuous variables
[e]SEP in 6 levels

For peer review only

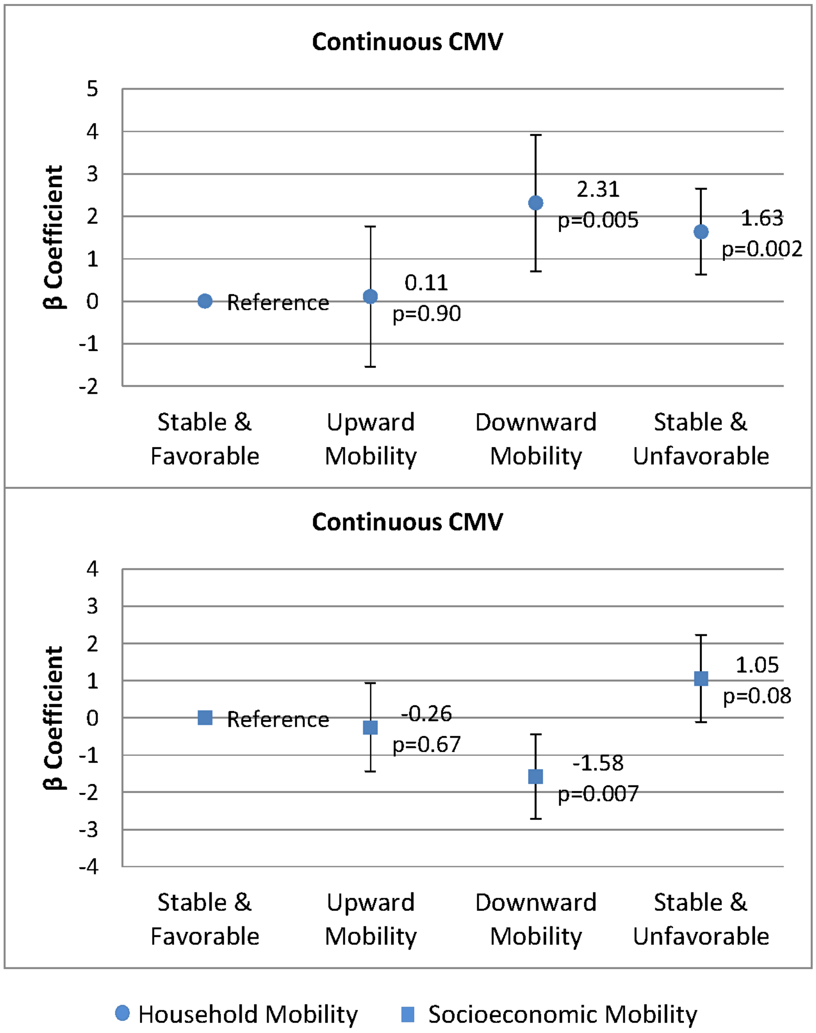

Supplement 7. Predicted mean anti-CMV IgG titer differences by household and socioeconomic mobility among the seropositive population. Predicted mean differences calculated via β coefficients from linear regression models adjusted for sex, maternal and paternal age at offspring birth and maternal, paternal and offspring smoking.

Associations of social environment, socioeconomic position and social mobility with immune response in young adults: The Jerusalem Perinatal Family Follow-up Study

List of Authors:

Gabriella M. Lawrence,[1] Yechiel Friedlander,[1] Ronit Calderon-Margalit,[1] Daniel A. Enquobahrie,[2,3] Jonathan Y. Huang,[3,4] Russell P. Tracy,[5] Orly Manor,[1] David S. Siscovick,[6] and Hagit Hochner[1]

[1]Braun School of Public Health, Hebrew University-Hadassah Medical Center, Jerusalem, Israel, [2]Cardiovascular Health Research Unit, [3]Department of Epidemiology, University of Washington, Seattle, WA,USA, [4]Institute for Health and Social Policy, McGill University, Montreal, QC, [5]Departments of Pathology and Biochemistry, University of Vermont, Burlington, VT, USA [6]New York Academy of Medicine, New York, NY, USA

Correspondence to:
Hagit Hochner, PhD
Unit of Epidemiology, Hebrew University-Hadassah Braun School of Public Health
POB 12272, Jerusalem 91120, Israel
Phone: +972-2-6777502 Fax: +972-2-6431086
Email: hagith@ekmd.huji.ac.il

Word Count: 3,233

For peer review only - http://bmjopen.bmj.com/site/about/guidelines.xhtml

ABSTRACT

Objectives: Immune response to cytomegalovirus (CMV) impacts adult chronic disease. This study investigates associations of childhood and adulthood social environment, socioeconomic position (SEP) and social mobility with CMV -response in young-adults.

Design: Historical prospective study design

Setting: Subcohort of all 17,003 births to residents of Jerusalem between 1974 and 1976.

Participants: Participants included 1,319 young adults born in Jerusalem with extensive archival and follow-up data, including childhood and adulthood SEP-related factors and anti-CMV IgG titer levels and seroprevalence measured at age 32.

Main exposure and outcome measures: Principal component analysis (PCA) was used to transform correlated social environment and SEP-related variables at two time points (childhood and adulthood) into two major scores reflecting household (e.g. number of siblings/children, religiosity) and socioeconomic (e.g. occupation, education) components. Based on these components, social mobility variables were created. Linear and Poisson regression models were used to investigate associations of components and mobility with anti-CMV IgG titer level and seroprevalence, adjusted for confounders.

Results: Lower levels of household and socioeconomic components in either childhood or adulthood were associated with higher anti-CMV IgG titer level and seropositivity at age 32. Compared to individuals with stable favorable components, anti-CMV IgG titer level and risk for seropositivity were higher in stable unfavorable household and socioeconomic components (household: β=3.23,p<0.001; RR=1.21,p<0.001; socioeconomic: β=2.20,p=0.001, RR=1.14,p=0.01), downward household mobility (β=4.32, p<0.001; RR=1.26, p<0.001) and upward socioeconomic mobility (β=1.37, p=0.04; RR=1.19, p<0.001). Among seropositive individuals, associations between household components and mobility with anti-CMV IgG titer level were maintained and associations between socioeconomic components and mobility with anti-CMV IgG titer level were attenuated.

For peer review only - http://bmjopen.bmj.com/site/about/guidelines.xhtml

Conclusions: Our study provides evidence that accumulating low SEP from childhood through adulthood and social mobility may compromise immune response in young adulthood

Keywords: Immune System, Epidemiology, Socioeconomic Position, Social Mobility, Cytomegalovirus, Cardiovascular

Strengths and limitations of this study

- Cytomegalovirus (CMV) antibody as a biomarker for atherosclerosis is a novel addition to studies of cardio-metabolic risk
- This is a unique population-based birth cohort with rich archival and follow-up data from birth to young adulthood
- We used an aggregate view of the social environment and SEP based on high-quality characterization in both childhood and adulthood to assess relationships at different time points in life as well as changes from childhood to adulthood with adult immune-response reflected by CMV titers
- Timing at initial seroconversion cannot be determined as only a single CMV titer measure was taken at young adulthood, also limiting precision in detection of lifetime viral load

review only

For peer review only - http://bmjopen.bmj.com/site/about/guidelines.xhtml

INTRODUCTION

Chronic inflammation is a known risk factor for cardiovascular disease, and infection-induced inflammation may be one of the causes of endothelial dysfunction leading to atherosclerosis[1]. Specifically, immune response to cytomegalovirus (CMV), a highly transmissible beta herpesvirus endemic throughout the world [2] and a biomarker of immune response [3,4], has been implicated in the development of atherosclerosis [3,5–7] and cardiovascular-related morbidity [8,9] and mortality[5,10–12]. Until recently, non-congenital CMV was not seen as clinically relevant in immunocompetent individuals due to the lack of overt clinical symptoms. However, accumulating evidence suggests that CMV antibody titer is associated with pro-inflammatory cytokines[3,4]; more memory T-cells are devoted to suppression of latent CMV [12,13] and fewer naïve T-cells are available to respond to new infections [12–14], leading to chronic immune activation and inflammation, and subsequent cardiovascular disease. Despite the sizeable cellular response from the host, a characteristic unique to CMV, the virus adapts to the immune system efficiently. CMV, therefore, is never eliminated from the infected immunocompetent individual, in whom it causes a persistent asymptomatic infection [15,16].

Research suggests that early-life environments affect immune function over the life-course[17]. Studies show that early-life stress results in infection-related premature death, immunosuppression [18], impairment in components of cell-mediated immunity, reduction in antibody response to vaccination and elevated antibody level [17,19,20] during childhood and beyond. A life-course approach to health proposes that interaction of environment and biological factors – e.g. epigenetic programming - experienced throughout the life-course impact current and future health[21–23].

For peer review only - http://bmjopen.bmj.com/site/about/guidelines.xhtml

Additionally, research has clearly indicated that socioeconomic factors throughout the life course impact adult health[21,24–30] and particularly in Israel, religious observance, a central characteristic of the Israeli social environment, contributes independently to social position and adult health [31]. Associations between CMV seropositivity and antibody titer with education, income [32], household crowding [2,33] and family size [34] further indicate that social factors influence immune response. Additionally, recent studies examining the impact of social mobility and socioeconomic gradients on adult health report varied results [24,25,35,36] . While upward mobility may attenuate the harmful effects of earlier disadvantage, and downward mobility is hypothesized to have a detrimental influence on health despite advantages at earlier ages, sustained exposure to low socioeconomic position (SEP) is most commonly associated with worse health [25,36,37]. Though associations between early-life socioeconomic factors and CMV have been demonstrated, the effect of social transitions from childhood to adulthood on CMV-response has not been investigated.

This study investigates associations between SEP in childhood and adulthood and social mobility from birth to 32 years with CMV-response in young-adults of the Jerusalem Perinatal Family Follow-up Study.

METHODS

The Jerusalem Perinatal Study (JPS) population-based cohort includes a subcohort of all 17,003 births to residents of Jerusalem between 1974 and 1976 [38,39]. Data consist of demographic and socioeconomic information, maternal medical conditions during current and previous pregnancies, and offspring birth-weight, abstracted from birth certificates or maternity ward logbooks. Additional information, including socioeconomic-related factors, gestational-age,

For peer review only - http://bmjopen.bmj.com/site/about/guidelines.xhtml

For peer review only

smoking, pre-pregnancy and end of pregnancy weight, and gynecological history, was collected by interviewing mothers on first or second postpartum day. Detailed information on data collection is previously described [38,39].

The JPS Family Follow-Up study includes a sample of 1,400 offspring from the 1974-1976 cohort interviewed and examined between 2007-2009 (mean age=32; range: 30-35 years) [40]. Briefly, sampling frame included singletons and term (gestational age ≥36 weeks) births without congenital malformations stratified by maternal pre-pregnancy BMI and birth-weight. Blood samples at fasting were taken and offspring IgG antibodies to CMV antigen were measured in plasma using indirect enzyme immunoassay with the Diamedix Immunosimplicity Is-CMV IgG Test Kit (Diamedex Corporation, 2008) at the University of Vermont. Further Test Kit details provided in supplement 1. Anti-CMV IgG titer levels were measured for 1,319 offspring with complete data.

This study was approved by the Institutional Review Board of the Hadassah-Hebrew University Medical Center and by the University of Washington Human Subject Review Committee. All participants provided informed consent.

Study Variables

Outcome Variables

The primary outcome examined was offspring CMV-response measured through offspring antibody to CMV at age 32. Transformation (square-root) of anti-CMV IgG titer level (EU/ml) was performed due to right skewness and treated as a continuous variable. CMV seroprevalence (seropositivity ≥10 EU/ml) was examined as a secondary dichotomous outcome.

For peer review only - http://bmjopen.bmj.com/site/about/guidelines.xhtml

Explanatory Variables

The multi-ethnic society of Israel[41] provides an opportunity to examine SEP alongside culturally relevant social environment characteristics, including religiosity and family-size in addition to well-recognized SEP variables such as occupation and education.

SEP-related explanatory variables were examined at birth and age 32, reflecting early (i.e., pre- and peri-natal periods) and young-adult life-course periods, respectively. Variables at childhood include (1)maternal religiosity (self-identified as ultra-orthodox, orthodox, traditional, secular and collapsed into dichotomous categories: ultra-orthodox and orthodox as religious, traditional and secular as not religious); (2)paternal lay-leadership (i.e. whether father was an ordained rabbi, dichotomous); (3)father's occupation (scale:1-6; 1-low, 6-high) as described by Corcoran[26]; (4)maternal education (years, continuous); and (5)number of siblings (continuous). Variables at adulthood include (1)offspring religiosity (self-identified as ultra-orthodox, orthodox, traditional, secular and collapsed into dichotomous categories: ultra-orthodox and orthodox as religious, traditional and secular as not religious); (2)offspring occupation (scale :1-6; 1-low, 6-high); (3)offspring education (years, continuous); and (4)parity at age 32 (continuous).

Potential confounders include offspring sex (dichotomous), maternal and paternal age at childbirth (years, continuous), maternal and paternal smoking during pregnancy and offspring smoking in young-adulthood (number of cigarettes smoked per day: 0, 1-10, 11-20, 21+).

Principal Component Analysis (PCA)

For peer review only - http://bmjopen.bmj.com/site/about/guidelines.xhtml

Following the successful use of PCA in creating socioeconomic indices in past and current studies[42,43], and given the available array of SEP-related variables, including SEP variables well known in the literature as well as specific social environment-related variables mentioned above, and the overlap and correlation between SEP-related variables (including individual-level indicators like education, occupation-based indicators and life course SEP)[44], we used PCA to reduce the number of potentially correlated variables in our cohort to a smaller number of principal components. (See Supplement 2 for correlations between SEP-related variables in the JPS Family Follow-up cohort.) PCA [45] carried out separately for variables measured at childhood and adulthood, transformed SEP-related variables into two major components: the social environment reflected by household-related characteristics (e.g. number of siblings/children, religiosity) and the socioeconomic (e.g. occupation, education) environment. The two components together explained 74% of childhood and 75% of adulthood variance. In addition to capturing the largest possible variance, these components nicely portray two elements of SEP in Israeli society – the socioeconomic environment represented by parental and offspring education and occupation as is often the case in many societies, and the social environment, unique to Israeli society, in which families that identify as more religious tend to have more children[46], with the most religious families often among those with lower income level[47]. Detailed information on component construction and variables included in PCA are provided in supplement 3. Higher component score indicates improvement in SEP – i.e. higher household component score indicates fewer children/decreased religiosity and higher socioeconomic component score indicates higher occupation level/years of education.

Social Mobility

For peer review only - http://bmjopen.bmj.com/site/about/guidelines.xhtml

For both household and socioeconomic components, childhood to adulthood social mobility variables were created. Components were divided into tertiles and dichotomous variables were created for each component (lowest tertile = low; middle and high tertile = high). Four categories of childhood to adulthood transition were created separately for household and socioeconomic component mobility: stable favorable (high-high), upward mobility (low-high), downward mobility (high-low), and stable unfavorable (low-low).

Statistical Analyses

Analysis of variance model, adjusted for sex, was used to compare mean anti-CMV IgG titer level by categories of characteristics obtained at birth and age 32. Linear regression models were used to investigate associations of childhood and adulthood household and socioeconomic components as well as social mobility with anti-CMV IgG titer level measured at age 32. Multivariable models were fitted to assess associations between childhood and adulthood household and socioeconomic components, independently of each other, with anti-CMV IgG titer controlling for sex, parental ages at offspring birth and parental and offspring smoking. Multivariable models were also fitted to examine associations between individual socioeconomic and household-related variables (i.e. father's occupation, maternal education, maternal religiosity, paternal lay leadership, number of siblings, offspring occupation, offspring education, offspring religiosity and parity), independently of each other, with anti-CMV IgG titer controlling for sex, parental ages at offspring birth and parental and offspring smoking. Similar multivariable models were fitted to assess associations between household and socioeconomic mobility with anti-CMV IgG titer controlling for sex, parental ages at offspring birth and parental and offspring smoking. Poisson regression models with robust error variance were used to

For peer review only - http://bmjopen.bmj.com/site/about/guidelines.xhtml

estimate relative risk of CMV seropositivity for each unit increase in component scores and among categories of social mobility variables. In order to verify that associations between socioeconomic factors and CMV titer levels are not primarily a reflection of associations between serostatus and CMV, all analyses were carried out for the total population (both seropositive and seronegative) and additionally among only the CMV seropositive population.

All models used inverse probability weighting to account for stratified sampling. Analyses were carried out using Stata 12.0 (StataCorp, College Station, TX).

RESULTS

Parental and offspring characteristics obtained at birth and offspring characteristics at age 32, including anti-CMV IgG titer, CMV seroprevalence and social mobility are listed in Table 1. Mean anti-CMV IgG titer and seropositivity were greater among females than males. Overall, 79.7% of the study population was seropositive for CMV. For social mobility, 7.2% of the population was categorized as downwardly mobile in household component and 15.2% experienced downward socioeconomic-mobility; 7.1% and 16.9% experienced upward household and socioeconomic mobility respectively.

Table 1. Study Characteristics at Birth and Age 32[a] * (N=1,319)

**Cytomegalovirus**		
Anti-CMV IgG titer level[b]		
Females	12.0	(7.9)
Males	9.5	(7.7)
Total	10.8	(7.9)
CMV Seropositivity[b] %		
Females	84.4	
Males	75.0	
Total	79.7	
**Characteristics obtained at birth**		
Maternal Religiosity %		

For peer review only - http://bmjopen.bmj.com/site/about/guidelines.xhtml

Ultra-Orthodox	18.4	
Orthodox	22.2	
Traditional	35.2	
Secular	24.2	
Paternal Lay Leadership %		
Yes	14.0	
No	86.0	
Paternal Occupation Level %		
Low	40.0	
Middle	23.3	
High	36.7	
Maternal Years of Education (years)	11.6	(3.4)
Number of Siblings %		
≤2	30.0	
3-4	39.5	
5+	30.5	
Maternal Age at Childbirth (years)	28.1	(5.6)
Paternal Age at Childbirth (years)	32.0	(6.9)
Maternal Smoking in Pregnancy %		
No	86.7	
Yes	13.3	
Paternal Smoking in Pregnancy %		
No	56.6	
Yes	43.4	
**Characteristics obtained at age 32**		
Religiosity %		
Ultra-Orthodox	20.5	
Orthodox	20.8	
Traditional	20.6	
Secular	38.1	
Occupation Level %		
Low	10.8	
Middle	33.2	
High	56.0	
Years of Education	15.5	(3.9)
Parity %		
≤2	62.6	
3-4	22.8	
5+	14.6	
Smoking %		
No	59.4	
Yes	40.6	
**Social Mobility**[d]		
Household[c] Mobility %		

For peer review only - http://bmjopen.bmj.com/site/about/guidelines.xhtml

Stable & Favorable	58.8
Upward Mobility	7.1
Downward Mobility	7.2
Stable & Unfavorable	26.9
Socioeconomic[c] Mobility %	
Stable & Favorable	49.5
Upward Mobility	16.9
Downward Mobility	15.2
Stable & Unfavorable	18.4

CMV = cytomegalovirus
[a]Values are expressed as mean (Standard Deviation) or percent
[b]obtained at age 32; square root transformed EU/ml; Seroprevalence = IgG titer level ≥10 EU/ml
[c]Household and Socioeconomic components created using principal component analysis detailed in methods; higher component score indicates improvement in household or socioeconomic factors
[d]Social mobility variables represent transition from childhood to adulthood using dichotomized household and socioeconomic components

Table 2 presents mean anti-CMV IgG titer level by categories of characteristics obtained at birth and age 32 as well as social mobility among the total population. Among the total population, mean anti-CMV IgG was highest among offspring born to ultra-orthodox mothers and fathers holding religious leadership positions, as well as offspring born to mothers with <9 years of education and those with ≥5 siblings. Similarly, ultra-orthodox offspring at age 32, those with ≤12 years of education and with more than 5 children had higher mean anti-CMV IgG titer level. Among CMV seropositive individuals, a similar pattern of mean anti-CMV IgG titer level by categories of characteristics obtained at birth and age 32 was found (supplement 4).

Table 2. Mean[a] Anti-CMV IgG Titer Level[b] by Categories of Characteristics Obtained at Birth and Age 32 (N=1,319)

	Mean	(SE)	P-value[e]
**Characteristics obtained at birth**			
Maternal Religiosity			<.0001
Ultra-Orthodox	12.9	(0.52)	
Orthodox	11.4	(0.47)	

For peer review only - http://bmjopen.bmj.com/site/about/guidelines.xhtml

Traditional	10.5	(0.37)	
Secular	8.5	(0.43)	
Paternal Lay-Leadership			<.0001
Yes	13.5	(0.23)	
No	10.3	(0.58)	
Paternal Occupation Level			0.8
Low	10.4	(0.34)	
Middle	10.6	(0.44)	
High	10.6	(0.35)	
Maternal Education			0.003
<9 years	11.9	(0.47)	
9-12 years	10.8	(0.33)	
>12 years	9.9	(0.36)	
Number of Siblings			<.0001
≤2	9.4	(0.38)	
3-4	10.3	(0.34)	
5+	12.8	(0.40)	
**Characteristics obtained at age 32**			
Religiosity			<0.0001
Ultra-Orthodox	12.6	(0.48)	
Orthodox	12.2	(0.48)	
Traditional	11.0	(0.48)	
Secular	9.1	(0.34)	
Occupation Level			0.01
Low	11.4	(0.68)	
Middle	11.6	(0.39)	
High	10.2	(0.29)	
Years of Education			0.001
≤ 12	11.9	(0.42)	
>12	10.3	(0.25)	
Parity			<0.0001
≤2	9.6	(0.27)	
3-4	12.5	(0.45)	
5+	13.1	(0.57)	

CMV = cytomegalovirus; SE = standard error

[a]Sex-adjusted

[b]square root transformed EU/ml

[c]Obtained through ANOVA

In order to model associations and adjust for additional covariates (i.e. offspring sex, parental ages at birth and parental and offspring smoking), we investigated associations between

For peer review only - http://bmjopen.bmj.com/site/about/guidelines.xhtml

household and socioeconomic components with anti-CMV IgG titer using linear regression models and with CMV seroprevalence using Poisson regression models presented in Table 3.

For peer review only

For peer review only - http://bmjopen.bmj.com/site/about/guidelines.xhtml

Table 3. Regression Coefficients, Relative Risks and 95% CI of CMV in Relation to Household and Socioeconomic Components[c] in Childhood and Adulthood

	Continuous CMV[a]						Seroprevalence[b]					
	Model 1[d]			Model 2[d]			Model 1[d]			Model 2[d]		
**Component**[c]	**Coef.**[d]	***P***	**95% CI**	**Coef.**[d]	***P***	**95% CI**	**RR**[d]	***P***	**95% CI**	**RR**[d]	***P***	**95% CI**
Household												
Childhood	-0.78	<0.001	-1.11,-0.45	-0.07	0.76	-0.53,0.39	0.96	0.001	0.94,0.98	1.00	0.86	0.97,1.04
Adulthood	-1.25	<0.001	-1.61,-0.90	-1.02	<0.001	-1.55,-0.49	0.94	<0.001	0.91,0.97	0.95	0.02	0.91,0.99
Socioeconomic												
Childhood	-1.04	<0.001	-1.43,-0.65	-0.74	0.001	-1.17,-0.31	0.91	<0.001	0.88,0.95	0.94	0.001	0.90,0.98
Adulthood	-0.71	<0.001	-1.09,-0.33	-0.39	0.08	-0.82,0.04	0.93	<0.001	0.90,0.97	0.95	0.02	0.92,0.99

Coef. = coefficient; CI = confidence interval; CMV = cytomegalovirus; RR = relative risk
[a]continuous square root transformed EU/ml
[b]Anti-CMV IgG titer level ≥10 EU/ml
[c]Created using principal component analysis detailed in methods
[d]Model 1 includes each component separately. Model 2 includes all four components together in model. All models adjusted for sex, maternal and paternal age at offspring birth and maternal, paternal and offspring smoking
[d]Coefficient = change in anti-CMV IgG titer level per increase in one unit of component (distribution characteristics in supplement 3); RR = change in risk estimate of CMV seropositivity per increase in one unit of component – calculated by Poisson regression with robust error variance

Lower levels of household and socioeconomic components in childhood and adulthood were associated with higher anti-CMV IgG titer level at age 32 (Table 3, continuous CMV Model 1) and increased CMV seropositivity (Table 3, seroprevalence Model 1). When childhood and adulthood components were included together in one model (Table 3, continuous CMV Model 2), only adulthood household and childhood socioeconomic components were associated with anti-CMV IgG titer (β= -1.02, p<0.001 and β= -0.74,

For peer review only - http://bmjopen.bmj.com/site/about/guidelines.xhtml

For peer review only

p=0.001, respectively). Among seropositive individuals, associations between household components and anti-CMV IgG titer level were maintained and associations between socioeconomic components and anti-CMV IgG titer level were attenuated (supplement 5). Models looking at associations between individual socioeconomic and household-related variables with anti-CMV IgG titer level found similar results to PCA-based models (supplement 6). We also investigated associations between household and socioeconomic component-mobility and anti-CMV IgG titer, adjusted for sex, parental ages at offspring birth and parental and offspring smoking (Figure 1). Compared to individuals with stable favorable household components, mean anti-CMV IgG levels were higher in those who maintained unfavorable household components from childhood to adulthood (β=3.23, $p<0.001$) and those who transitioned from a favorable to unfavorable position (i.e. downward mobility) (β=4.32, $p<0.001$). Those who maintained a stable unfavorable socioeconomic component position had increased anti-CMV titer level compared to those in the stable favorable position (β=2.20, p=0.001) as did those in the upward mobility category (β=1.37, p=0.04). A similar pattern of associations was seen when investigating associations between mobility and CMV seroprevalence (Figure 1). To distinguish mobility effects from main effects of childhood and adulthood components, each mobility model was further adjusted – once for the respective (household or socioeconomic) childhood component and separately for the respective adulthood component. Associations between household mobility and anti-CMV titer level as well as with seroprevalence were maintained, while associations between socioeconomic mobility and anti-CMV titer level as well as with seroprevalence were attenuated when the models were adjusted for the socioeconomic childhood component (data not shown). Among

For peer review only - http://bmjopen.bmj.com/site/about/guidelines.xhtml

CMV seropositive individuals, similar associations were found between household component mobility and anti-CMV IgG titer and associations between socioeconomic component mobility and anti-CMV IgG titer level were primarily attenuated (supplement 7).

DISCUSSION

This study demonstrated that lower levels of household and socioeconomic components in childhood and adulthood, reflecting lower SEP, were associated with higher anti-CMV IgG titer level at age 32. Additionally, maintaining a stable unfavorable position from childhood to adulthood was associated with higher anti-CMV IgG titer level compared to the stable favorable position. Our study also provides some evidence that social mobility transitions from childhood to adulthood (i.e. upward and downward mobility) may be associated with CMV-response.

Life-Course Models

Life-course models explaining the impact of SEP on adult health proposed in the literature include the critical period model, emphasizing timing of exposure[48,49], the social mobility model, focusing on transitions across the life-course[48,49], and the accumulation model, emphasizing duration of exposures[48,49]. As per the critical period model, our findings that lower childhood and adulthood household and socioeconomic components are associated with higher anti-CMV IgG titer level in early adulthood are in accordance with other studies[17,27]. Our study also adds to the social mobility model, though unlike others[24,28,35], we found that upward socioeconomic mobility was associated with higher CMV titer and higher risk for CMV seropositivity. Possibly, as others, including JPS analyses, have found[29], mechanisms similar to those in Gluckman's "Mismatch hypothesis” are at play – in which early environment

For peer review only - http://bmjopen.bmj.com/site/about/guidelines.xhtml

induces long-term cardiovascular effects if there is a "mismatch" (including improvement) between early developmental environment and subsequent environment in later life. Additionally, the attenuation of the associations between upward socioeconomic mobility and CMV with further adjustment for the socioeconomic component at baseline suggests that the socioeconomic environment follows a critical period model as opposed to a social mobility model. Household downward mobility, as in other cohort-based research[25], was associated with increased CMV IgG titer levels and increased risk for CMV seropositivity, even after adjustment for household components at childhood or adulthood, suggesting that the household environment has a mobility effect above and beyond that of a critical period. Our study is the first to investigate social mobility associations with young-adult CMV-response.

Research has indicated that sustained exposure to low SEP has been most commonly associated with worse health[25,28,35–37,48]. Our study supports the detrimental impact of accumulation of lower SEP over the life-course, as we found increased anti-CMV IgG titer and risk for CMV seropositivity among those in both the household and socioeconomic stable unfavorable group as compared to stable favorable.

Multidimensionality of Exposure

The associations of social mobility with young-adult CMV-response differ somewhat in household and socioeconomic components. While household downward mobility was associated with increased anti-CMV IgG titer level, upward socioeconomic mobility seemingly had a harmful impact on immune response at age 32. These findings highlight the multidimensionality of socioeconomic measurement and the varying contribution of different

For peer review only - http://bmjopen.bmj.com/site/about/guidelines.xhtml

socioeconomic dimensions through the life-course[30]. The PCA used in our analyses may provide a more aggregate characterization of the socioeconomic experience/environment, while demonstrating that various socioeconomic factors may indeed represent different domains. To further highlight this point, household upward or downward mobility comprised only 14.3% of the study population while socioeconomic mobility was seen among 31.1%, possibly indicating that the household component is more stable than the socioeconomic component. Possibly, people tend to maintain a level of religiosity similar to that with which they were raised[50] whereas socioeconomic transitions from parents to offspring vary depending on culture, environment, psychological elements and geographic residence[51].

Epigenetic Mechanism

Previous studies found that participants originating from low and high SEP have different patterns of methylation[52]. Additionally, associations have been found between methylation and chronic inflammation and a previous analysis of JPS demonstrated an *HSD11B2*–cardiometabolic risk association[23]. Epigenetic changes in response to environment may play an important role in the development of adult diseases[53]. Further studies that investigate an epigenetic role in the SEP–CMV associations may provide evidence for mechanisms that explain these associations.

Study Strengths and Limitations

One of the major strengths of this study is the novel use of CMV antibody, emerging as a potential biomarker for atherosclerosis and cardiometabolic risk, as well as the use of continuous CMV antibody in addition to CMV seropositivity as a study outcome. Few studies

For peer review only - http://bmjopen.bmj.com/site/about/guidelines.xhtml

have examined CMV antibody titer on a continuous scale, despite potential epidemiological significance of such an outcome. Additionally, very few studies have had the ability to examine the impact of SEP and social mobility on immune-response in young adulthood. The combination of high-quality detailed records of perinatal maternal and offspring characteristics and long-term follow-up data at age 32, improved the characterization of the socioeconomic environment surrounding pregnancy, childhood and adulthood, and allowed analyses to examine changes from childhood to adulthood. Estimating different components of the socioeconomic environment and trajectory by PCA was another novel approach, enabling us to capture common variance and providing an aggregate view of the socioeconomic experience/environment, rather than limiting analyses to individual SEP proxies.

Our study has several limitations. First, it includes only a sample of offspring from the original 1974-1976 JPS cohort invited to participate in the follow-up study. However, using a stratified sampling approach and over-sampling ends of the distribution ensured that offspring with a range of fetal and early-life characteristics were included in our study. Second, examining CMV titer on a continuous scale using qualitative ELISA assay limits clinical interpretation of findings. However, our findings using CMV seropositivity, a clinically relevant diagnosis, support the associations found with CMV antibody titer, and substantiate the use of continuous CMV antibody in future epidemiological studies. Importantly, this specific assay was successfully used in other cohorts, such as the Multi-Ethnic Study of Atherosclerosis[12]. It is important to note that while the immune response to CMV has an extensive impact on the immune system, using CMV antibody titer as a proxy measure of the general cellular immune condition may not be ideal, particularly given the unique cellular response to CMV from the host. More modern

For peer review only - http://bmjopen.bmj.com/site/about/guidelines.xhtml

approaches, using multiple technological platforms, where the multiple responding cells and cytokines in the blood can be measured[54], may give a more in-depth understanding of the overall immune response. Additionally, data consists of a single CMV titer measure for each participant. Verification of titer level from additional tests or other sources was not available, and timing/age at initial seroconversion is unknown. However, research has indicated that CMV antibody titer remains relatively stable over time[55,56], and seroprevalence rates in our population fall in the range of prevalence rates in similar populations[2,15,16,57]. Furthermore, the use of PCA may mask distinctions in relationships between individual SEP variables and CMV. However, additional analyses revealed that associations with the individual SEP variables are in line with what has been previously reported in the literature[32] and similar to PCA-based results, found negative associations between education and anti-CMV IgG titer level, particularly in childhood and positive associations between religiosity and family size with anti-CMV IgG titer particularly in adulthood. PCA was used to reduce the number of potentially correlated variables in our cohort to a smaller number of principal components that would produce a more coherent picture of the SEP-CMV relationship. While relationships between SEP and CMV have been reported in studies in other populations, the differing associations found in our study between socioeconomic vs. household components, may be particular to unique elements in Israeli society.

Conclusions

Our study extends accumulating evidence of relationships between socioeconomic factors in childhood and adulthood with adult immune response. Additionally, our study provides

For peer review only - http://bmjopen.bmj.com/site/about/guidelines.xhtml

evidence that accumulating low SEP from childhood through adulthood and social mobility may compromise immune response in young adulthood. Further studies should explore mechanisms underlying the relationships between childhood environments and social mobility with immunity in adulthood. A better understanding of these relationships may lead to new approaches to counteract effects of low-SEP environments through the life-course and improve immune response in high-risk young-adults.

For peer review only

REFERENCES

1 Ross R. Inflammation or Atherogenesis. *N Engl J Med* 1999;**340**:115–26. doi:10.1056/NEJM199901143400207

2 Bate SL, Dollard SC, Cannon MJ. Cytomegalovirus seroprevalence in the United States: the national health and nutrition examination surveys, 1988-2004. *Clin Infect Dis* 2010;**50**:1439–47. doi:10.1086/652438

3 Nazmi A, Diez-Roux A V, Jenny NS, *et al.* The influence of persistent pathogens on circulating levels of inflammatory markers: a cross-sectional analysis from the Multi-Ethnic Study of Atherosclerosis. *BMC Public Health* 2010;**10**:706. doi:10.1186/1471-2458-10-706

4 Marashi SM, Raeiszadeh M, Enright V, *et al.* Influence of cytomegalovirus infection on immune cell phenotypes in patients with common variable immunodeficiency. *J Allergy Clin Immunol* 2012;**129**:1349–1356.e3. doi:10.1016/j.jaci.2012.02.011

5 Simanek AM, Dowd JB, Pawelec G, *et al.* Seropositivity to cytomegalovirus, inflammation, all-cause and cardiovascular disease-related mortality in the United States. *PLoS One* 2011;**6**:e16103. doi:10.1371/journal.pone.0016103

6 Zhu J, Quyyumi A a, Norman JE, *et al.* Cytomegalovirus in the pathogenesis of atherosclerosis. *J Am Coll Cardiol* 1999;**34**:1738–43. doi:10.1016/S0735-1097(99)00410-6

7 Blum a., Peleg a., Weinberg M. Anti-cytomegalovirus (CMV) IgG antibody titer in patients with risk factors to atherosclerosis. *Clin Exp Med* 2003;**3**:157–60. doi:10.1007/s10238-003-0019-7

8 Ji Y-N, An L, Zhan P, *et al.* Cytomegalovirus infection and coronary heart disease risk: a meta-analysis. *Mol Biol Rep* 2012;**39**:6537–46. doi:10.1007/s11033-012-1482-6

9 Zhang M, Yang Y, Yang X, *et al.* Human cytomegalovirus infection is a novel etiology for essential hypertension. *Med Hypotheses* 2011;**76**:682–4. doi:10.1016/j.mehy.2011.01.032

10 Roberts ET, Haan MN, Dowd JB, *et al.* Cytomegalovirus antibody levels, inflammation, and mortality among elderly Latinos over 9 years of follow-up. *Am J Epidemiol* 2010;**172**:363–71. doi:10.1093/aje/kwq177

11 Strandberg TE, Pitkala KH, Tilvis RS. Cytomegalovirus antibody level and mortality among community-dwelling older adults with stable cardiovascular disease. *JAMA* 2009;**301**:380–2. doi:10.1001/jama.2009.4

12 Tracy RP, Doyle MF, Olson NC, *et al.* T-helper type 1 bias in healthy people is associated with cytomegalovirus serology and atherosclerosis: the Multi-Ethnic Study of Atherosclerosis. *J Am Heart Assoc* 2013;**2**:e000117. doi:10.1161/JAHA.113.000117

13 Olson NC, Doyle MF, Jenny NS, *et al.* Decreased naive and increased memory CD4(+) T cells are associated with subclinical atherosclerosis: the multi-ethnic study of atherosclerosis. *PLoS One* 2013;**8**:e71498. doi:10.1371/journal.pone.0071498

14 Zhu J. Discordant cellular and humoral immune responses to cytomegalovirus infection in

For peer review only - http://bmjopen.bmj.com/site/about/guidelines.xhtml

healthy blood donors: existence of a Th1-type dominant response. *Int Immunol* 2001;**13**:785–90. doi:10.1093/intimm/13.6.785

15 La Rosa C, Diamond DJ. The immune response to human CMV. *Future Virol* 2012;**7**:279–93. doi:10.2217/fvl.12.8

16 Levine H, Balicer RD, Rozhavski V, *et al.* Seroepidemiology of Epstein-Barr virus and cytomegalovirus among Israeli male young adults. *Ann Epidemiol* 2012;**22**:783–8. doi:10.1016/j.annepidem.2012.06.099

17 McDade TW. Life history, maintenance, and the early origins of immune function. *Am J Hum Biol* 2005;**17**:81–94. doi:10.1002/ajhb.20095

18 Moore SE, Cole TJ, Collinson AC, *et al.* Prenatal or early postnatal events predict infectious deaths in young adulthood in rural Africa. *Int J Epidemiol* 1999;**28**:1088–95.

19 Shirtcliff EA, Coe CL, Pollak SD. Early childhood stress is associated with elevated antibody levels to herpes simplex virus type 1. *Proc Natl Acad Sci U S A* 2009;**106**:2963–7. doi:10.1073/pnas.0806660106

20 McDade TW, Stallings JF, Angold A, *et al.* Epstein-Barr virus antibodies in whole blood spots: a minimally invasive method for assessing an aspect of cell-mediated immunity. *Psychosom Med* 2000;**62**:560–7.

21 Power C, Atherton K, Strachan DP, *et al.* Life-course influences on health in British adults: effects of socio-economic position in childhood and adulthood. *Int J Epidemiol* 2007;**36**:532–9. doi:10.1093/ije/dyl310

22 Borghol N, Suderman M, McArdle W, *et al.* Associations with early-life socio-economic position in adult DNA methylation. *Int J Epidemiol* 2012;**41**:62–74. doi:10.1093/ije/dyr147

23 Huang JY, Gavin AR, Richardson TS, *et al.* Accounting for life course exposures in epigenetic biomarker association studies: Early life socioeconomic position, candidate gene DNA methylation, and adult cardiometabolic risk. *Am J Epidemiol* 2016;**in-press**.

24 Aitsi-Selmi A, Batty GD, Barbieri MA, *et al.* Childhood socioeconomic position, adult socioeconomic position and social mobility in relation to markers of adiposity in early adulthood: evidence of differential effects by gender in the 1978/79 Ribeirao Preto cohort study. *Int J Obes (Lond)* 2013;**37**:439–47. doi:10.1038/ijo.2012.64

25 Smith BT, Lynch JW, Fox CS, *et al.* Life-course socioeconomic position and type 2 diabetes mellitus: The Framingham Offspring Study. *Am J Epidemiol* 2011;**173**:438–47. doi:10.1093/aje/kwq379

26 Corcoran C, Perrin M, Harlap S, *et al.* Effect of socioeconomic status and parents' education at birth on risk of schizophrenia in offspring. Soc. Psychiatry Psychiatr. Epidemiol. 2009;**44**:265–71. doi:10.1007/s00127-008-0439-5

27 Demakakos P, Marmot M, Steptoe A. Socioeconomic position and the incidence of type 2 diabetes: The ELSA study. *Eur J Epidemiol* 2012;**27**:367–78. doi:10.1007/s10654-012-9688-4

For peer review only - http://bmjopen.bmj.com/site/about/guidelines.xhtml

28 Albrecht SS, Gordon-Larsen P. Socioeconomic gradients in body mass index (BMI) in US immigrants during the transition to adulthood: examining the roles of parental education and intergenerational educational mobility. *J Epidemiol Community Health* 2014;**68**:842–8. doi:10.1136/jech-2014-203814

29 Savitsky B, Manor O, Friedlander Y, *et al.* Associations of socioeconomic position in childhood and young adulthood with cardiometabolic risk factors: the Jerusalem Perinatal Family Follow-Up Study. *J Epidemiol Community Health* 2017;**71**:43–51. doi:10.1136/jech-2014-204323

30 Cutler DM, Lleras-Muney A VT. Socioeconomic status and health: Dimensions and mechanisms. *Oxford Handb Heal Econ* 2011;:124–163.

31 Kark JD, Shemi G, Friedlander Y, *et al.* Does Religious Observance Promote Health ? Mortality in Secular vs Religious Kibbutzim in Israel. *Am J Public Health* 1996;**86**:341–6.

32 Dowd JB, Aiello AE. Socioeconomic differentials in immune response. *Epidemiology* 2009;**20**:902–8. doi:10.1097/EDE.0b013e3181bb5302

33 Jansen MAE, van den Heuvel D, Bouthoorn SH, *et al.* Determinants of Ethnic Differences in Cytomegalovirus, Epstein-Barr Virus, and Herpes Simplex Virus Type 1 Seroprevalence in Childhood. *J Pediatr* 2016;**170**:126–34. doi:10.1016/j.jpeds.2015.11.014

34 Shigemi D, Yamaguchi S, Otsuka T, *et al.* Seroprevalence of cytomegalovirus IgG antibodies among pregnant women in Japan from 2009-2014. *Am J Infect Control* 2015;**43**:1218–21. doi:10.1016/j.ajic.2015.06.026

35 Johnson-Lawrence V, Kaplan G, Galea S. Socioeconomic mobility in adulthood and cardiovascular disease mortality. *Ann Epidemiol* 2013;**23**:167–71. doi:10.1016/j.annepidem.2013.02.004

36 Heraclides A, Brunner E. Social mobility and social accumulation across the life course in relation to adult overweight and obesity: the Whitehall II study. *J Epidemiol Community Health* 2010;**64**:714–9. doi:10.1136/jech.2009.087692

37 Halonen JI, Stenholm S, Pentti J, *et al.* Childhood Psychosocial Adversity and Adult Neighborhood Disadvantage as Predictors of Cardiovascular Disease: A Cohort Study. *Circulation* 2015;**132**:371–9. doi:10.1161/CIRCULATIONAHA.115.015392

38 Davies AM, Prywes R, Tzur B, *et al.* The Jerusalem perinatal study. 1. Design and organization of a continuing, community-based, record-linked survey. *Isr J Med Sci* 1969;**5**:1095–106.

39 Harlap S, Davies A, Deutsch L, *et al.* The Jerusalem Perinatal Study cohort, 1964-2005: methods and a review of the main results. *Paediatr Perinat Epidemiol* 2007;**21**:256–73. doi:10.1111/j.1365-3016.2007.00799.x.The

40 Lawrence GM, Siscovick DS, Calderon-Margalit R, *et al.* Cohort Profile: The Jerusalem Perinatal Family Follow-Up Study. *Int J Epidemiol* 2016;**45**:343–52. doi:10.1093/ije/dyv120

41 Haj M Al. *Immigration and Ethnic Formation in a Deeply Divided Society: The Case of the*

For peer review only - http://bmjopen.bmj.com/site/about/guidelines.xhtml

1990s Immigrants from the Former Soviet Union in Israel. BRILL 2004.

42 Vyas S, Kumaranayake L. Constructing socio-economic status indices: How to use principal components analysis. *Health Policy Plan* 2006;**21**:459–68. doi:10.1093/heapol/czl029

43 Becher H, Palm F, Aigner A, *et al.* Socioeconomic conditions in childhood, adolescence, and adulthood and the risk of ischemic stroke. *Stroke* 2016;**47**:173–9. doi:10.1161/STROKEAHA.115.011523

44 Galobardes B, Lynch J, Smith GD. Measuring socioeconomic position in health research. *Br Med Bull* 2007;**81**–**82**:21–37. doi:10.1093/bmb/ldm001

45 Stevens J. *Applied multivariate statistics for the social sciences*. L. Erlbaum Associates Inc. 1986.

46 Bystrov E. Religiosity, nationalism and fertility among Jews in Israel revisited. *Acta Sociol* 2016;**59**:171–86. doi:10.1177/0001699316628615

47 Bleikh H, Berrebi C, Brand G, *et al.* Poverty and Inequality in Israel: Trends and Decompositions. In: Weiss A, ed. *State of the Nation Report*. 2016. 315–51.

48 Pollitt RA, Rose KM, Kaufman JS. Evaluating the evidence for models of life course socioeconomic factors and cardiovascular outcomes: a systematic review. *BMC Public Health* 2005;**5**:7. doi:10.1186/1471-2458-5-7

49 Kuh D, Ben-Shlomo Y, Lynch J, *et al.* Life course epidemiology. *J Epidemiol Community Health* 2003;**57**:778–83.

50 Ashton MC. *Individual Differences and Personality*. Academic Press 2013.

51 *From Parents to Children: The Intergenerational Transmission of Advantage*. Russell Sage Foundation 2012.

52 Lam LL, Emberly E, Fraser HB, *et al.* Factors underlying variable DNA methylation in a human community cohort. *Proc Natl Acad Sci U S A* 2012;**109 Suppl**:17253–60. doi:10.1073/pnas.1121249109

53 Gilbert ER, Liu D. Epigenetics: the missing link to understanding β-cell dysfunction in the pathogenesis of type 2 diabetes. *Epigenetics* 2012;**7**:841–52. doi:10.4161/epi.21238

54 Furman D, Davis MM. New approaches to understanding the immune response to vaccination and infection. *Vaccine* 2015;**33**:5271–81. doi:10.1016/j.vaccine.2015.06.117

55 Zanghellini F, Boppana SB, Emery VC, *et al.* Asymptomatic primary cytomegalovirus infection: virologic and immunologic features. *J Infect Dis* 1999;**180**:702–7. doi:10.1086/314939

56 Miller MS, Gardner TJ, Krammer F, *et al.* Neutralizing antibodies against previously encountered influenza virus strains increase over time: a longitudinal analysis. *Sci Transl Med* 2013;**5**:198ra107. doi:10.1126/scitranslmed.3006637

57 Cannon MJ, Schmid DS, Hyde TB. Review of cytomegalovirus seroprevalence and demographic characteristics associated with infection. *Rev Med Virol* 2010;**20**:202–13.

For peer review only - http://bmjopen.bmj.com/site/about/guidelines.xhtml

doi:10.1002/rmv

For peer review only

For peer review only - http://bmjopen.bmj.com/site/about/guidelines.xhtml

Contributors: DSS, HH and YF conceived and designed the study. HH designed data collection tools and developed protocols.GML designed the analysis plan, conducted the analysis and wrote the manuscript with guidance from YF and HH. DSS and RPT advised on the clinical context of the study and analyses.OM, RC-M, DAE and JYH advised on data analysis and interpretation of findings. All authors contributed intellectually to the manuscript, reviewed and edited the manuscript and approved the final version.

Funding: This work was financially supported by the National Institutes of Health [grant numbers R01HL088884 and K01HL103174] and the Israeli Science Foundation [grant numbers 1252/07 and 552/12].

Disclaimer: The founders had no role in the design, analysis or writing of this article

Competing interests: None declared

Informed Consent: Informed consent was obtained from all individual participants included in the study.

Ethical Approval: This study was approved by the Institutional Review Board of the Hadassah-Hebrew University Medical Center and by the University of Washington Human Subject Review Committee.

Data sharing statement: Data are available on request. Email Dr. Hagit Hochner: hagith@ekmd.huji.ac.il

Figure Legend

Fig. Predicted mean anti-CMV IgG titer differences and CMV seropositivity risk by household and socioeconomic mobility. Predicted mean differences calculated via β coefficients from linear regression models adjusted for sex, maternal and paternal age at offspring birth and maternal, paternal and offspring smoking. Relative risk estimation by Poisson regression with robust error variance models adjusted for sex, maternal and paternal age at offspring birth and maternal, paternal and offspring smoking**.**

For peer review only

For peer review only - http://bmjopen.bmj.com/site/about/guidelines.xhtml

Supplement 1. Diamedix Immunosimplicity Is-CMV IgG Test Kit

(Diamedex Corporation, 2008)

The Test Kit is intended for the qualitative and semi-quantitative detection of antibodies to CMV antigen. Compared to currently used testing methods in three different United States-based laboratories, the sensitivity and specificity of the Diamedix Kit ranged from 99.3%-100.0% and 87.7%-100.0% respectively, and overall agreement ranged from 97.0%-99.0%. Seroprevalence results detected using this kit were in agreement with prevalence rates reported in United States blood donor populations.

Diamedix. CMV IgG: Enzyme Immunoassay Test Kit. 2008;3–6.

For peer review only

Supplement 2. Correlations between SEP-related variables in the JPS Family Follow-up cohort

	Father's occupation[a]	Maternal education[b]	Maternal religiosity[c]	Paternal lay-leadership[c]	Number of siblings[b]	Offspring occupation[a]	Offspring education[b]	Offspring religiosity[c]	Offspring parity[b]
Father's occupation[a]	1.00								
Maternal education[b]	0.52	1.00							
Maternal religiosity[c]	0.23	0.07	1.00						
Paternal lay-leadership[c]	0.32	0.10	0.49	1.00					
Number of siblings[b]	0.10	-0.14	0.54	0.60	1.00				
Offspring occupation[a]	0.22	0.29	0.00	0.02	-0.10	1.00			
Offspring education[b]	0.30	0.32	0.19	0.22	0.08	0.38	1.00		
Offspring religiosity[c]	0.19	0.06	0.72	0.47	0.53	0.00	0.18	1.00	
Offspring parity[b]	0.14	-0.04	0.50	0.49	0.54	-0.05	0.06	0.57	1.00

[a]In 6 categories; Spearman's rank correlation coefficient
[b]Continuous; Pearson correlation coefficient
[c]Dichotomous; Pearson correlation coefficient

For peer review only

Supplement 3. Principal Component Analysis Loadings

Childhood			Adulthood		
	Component Loadings			Component Loadings	
Variables Included	Household	Socioeconomic	Variables Included	Household	Socioeconomic
Paternal Occupation	0.112	0.656	Offspring Occupation	-0.111	0.719
Maternal Education	-0.132	0.723	Offspring Education	0.115	0.690
Maternal Religiosity	0.538	0.048	Offspring Religiosity	0.695	0.056
Paternal Lay Leadership	0.550	0.119			
Number of Siblings	0.616	-0.180	Offspring Parity	0.701	-0.065
Variance Explained	74%		Variance Explained	75%	

Principal Component Score Distribution

	Mean	SD	Minimum	Maximum
**Household Components**				
Childhood	-0.003	1.49	-4.63	1.86
Adulthood	-0.008	1.27	-4.05	2.08
**Socioeconomic Components**				
Childhood	0.04	1.21	-4.14	3.52
Adulthood	0.06	1.15	-3.66	3.02

For peer review only

Supplement 4. Mean[a] Anti-CMV IgG Titer Level[b] by Categories of Characteristics Obtained at Birth and Age 32 Among CMV seropositive individuals (N=1,051)

	Mean	(SE)	P-value[e]
**Characteristics obtained at birth**			
Maternal Religiosity			0.007
Ultra-Orthodox	14.34	(6.29)	
Orthodox	13.69	(7.09)	
Traditional	12.78	(6.34)	
Secular	12.37	(5.90)	
Paternal Lay-Leadership			0.06
Yes	14.12	(6.06)	
No	13.09	(6.52)	
Paternal Occupation Level			0.69
Low	13.11	(6.69)	
Middle	13.14	(6.53)	
High	13.48	(6.14)	
Maternal Education			0.26
<9 years	13.82	(7.02)	
9-12 years	13.07	(6.35)	
>12 years	13.00	(6.08)	
Number of Siblings			0.003
≤2	13.02	(6.48)	
3-4	12.92	(6.46)	
5+	13.72	(6.30)	
**Characteristics obtained at age 32**			
Religiosity			0.03
Ultra-Orthodox	13.70	(6.04)	
Orthodox	14.00	(7.08)	
Traditional	13.12	(6.45)	
Secular	12.52	(6.20)	
Occupation Level			0.06
Low	12.56	(6.12)	
Middle	13.90	(7.02)	
High	13.01	(6.12)	
Years of Education			0.32
≤ 12	13.53	(6.63)	
>12	13.10	(6.33)	
Parity			0.008
≤2	12.75	(6.44)	
3-4	13.73	(6.70)	
5+	14.07	(5.69)	

CMV = cytomegalovirus; SE = standard error
[a]Sex-adjusted
[b]square root transformed EU/ml
[c]Obtained through ANOVA

For peer review only

Supplement 5. Regression Coefficients and 95% CI of CMV in Relation to Household and Socioeconomic Components[b] in Childhood and Adulthood Among CMV Seropositive Population

	Continuous CMV[a]					
	Model 1[c]			Model 2[c]		
**Component[b]**	**Coef.[d]**	***P***	**95% CI**	**Coef.[d]**	***P***	**95% CI**
Household						
Childhood	-0.41	0.008	-0.71, -0.11	-0.03	0.91	-0.44, 0.39
Adulthood	-0.74	<0.001	-1.07, -0.42	-0.67	0.007	-1.15, -0.18
Socioeconomic						
Childhood	-0.23	0.24	-0.61, 0.15	-0.21	0.31	-0.63, 0.20
Adulthood	-0.01	0.94	-0.35, 0.38	-0.17	0.42	-0.25, 0.59

Coef. = coefficient; CI = confidence interval; CMV = cytomegalovirus
[a]Continuous square root transformed EU/ml
[b]Created using principal component analysis detailed in methods
[c]Model 1 includes each component separately. Model 2 includes all four components together in model. All models adjusted for sex, maternal and paternal age at offspring birth and maternal, paternal and offspring smoking
[d]Coefficient = change in anti-CMV IgG titer level per increase in one unit of component (distribution characteristics in supplement 3)

For peer review only

Supplement 6. Associations between social environment and socioeconomic factors with CMV antibody in total and CMV seropositive population

	Among Total Population						Among CMV Seropositive Only					
	Univariate Model[a]			Multivariate Model[b]			Univariate Model[a]			Multivariate Model[b]		
	B	P-value	95% CI	β	P-value	95% CI	B	P-value	95% CI	β	P-value	95% CI
**Characteristics at birth**												
Maternal education (yrs)[d]	-0.32	<0.001	-0.45, -0.19	-0.35	<0.001	-0.52, -0.19	-0.11	0.08	-0.23, 0.01	-0.22	0.007	-0.37, -0.06
Maternal religiosity[c]	2.28	<0.001	1.39, 3.17	-0.47	0.51	-1.89, 0.94	1.17	0.005	0.35, 1.99	-0.30	0.65	-1.59, 0.99
Paternal lay leadership[c]	2.40	<0.001	1.09, 3.71	-0.82	0.39	-2.70, 1.05	0.88	0.13	-0.26, 2.04	-0.47	0.59	-2.18, 1.24
Paternal SEP[d]	-0.09	0.46	-0.34, 0.15	0.16	0.31	-0.15, 0.48	0.12	0.31	-0.11, 0.35	0.22	0.15	-0.08, 0.54
Family size[d]	0.51	<0.001	0.34, 0.67	0.18	0.16	-0.07, 0.44	0.16	0.04	0.007, 0.30	0.01	0.91	-0.22, 0.25
**Characteristics at age 32**												
Education (yrs)[d]	-0.15	0.01	-0.27, -0.04	-0.07	0.36	-0.21, 0.08	0.01	0.81	-0.10, 0.12	0.10	0.18	-0.04, 0.24
Religiosity[c]	2.74	<0.001	1.89, 3.60	2.24	0.002	0.82, 3.66	1.55	<0.001	0.77, 2.34	1.88	0.004	0.60, 3.17
Current Family size[d]	0.79	<0.001	0.58, 1.00	0.22	0.15	-0.08, 0.52	0.28	0.005	0.08, 0.48	-0.13	0.37	-0.42, 0.16
Current SEP[d]	-0.01	0.92	-0.31, 0.28	0.09	0.58	-0.24, 0.43	0.20	0.15	-0.07, 0.48	0.08	0.64	-0.24, 0.39

[a]Univariate model includes each variable alone in model

[b]Multivariate model includes all variables together in one model + offspring sex, maternal and paternal age at childbirth, maternal and paternal smoking during pregnancy and offspring smoking in young-adulthood

[c]Treated as dichotomous variables

[d]Treated as continuous variables

[e]SEP in 6 levels

For peer review only

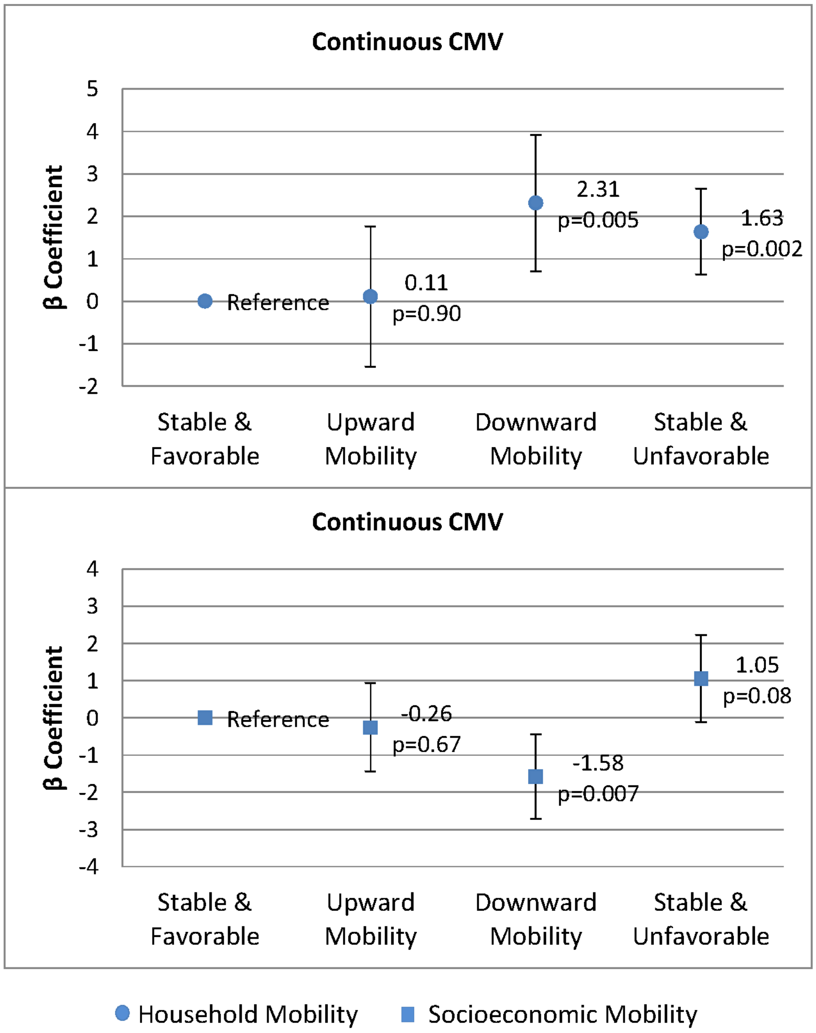

Supplement 7. Predicted mean anti-CMV IgG titer differences by household and socioeconomic mobility among the seropositive population. Predicted mean differences calculated via β coefficients from linear regression models adjusted for sex, maternal and paternal age at offspring birth and maternal, paternal and offspring smoking.

STROBE Statement—Checklist of items that should be included in reports of ***cohort studies***

	Item No	Recommendation	Page #
**Title and abstract**	1	(*a*) Indicate the study's design with a commonly used term in the title or the abstract	1
		(*b*) Provide in the abstract an informative and balanced summary of what was done and what was found	1
**Introduction**			
Background/rationale	2	Explain the scientific background and rationale for the investigation being reported	3-4
Objectives	3	State specific objectives, including any prespecified hypotheses	4
**Methods**			
Study design	4	Present key elements of study design early in the paper	4-5
Setting	5	Describe the setting, locations, and relevant dates, including periods of recruitment, exposure, follow-up, and data collection	4-5
Participants	6	(*a*) Give the eligibility criteria, and the sources and methods of selection of participants. Describe methods of follow-up	5
		(*b*) For matched studies, give matching criteria and number of exposed and unexposed	
Variables	7	Clearly define all outcomes, exposures, predictors, potential confounders, and effect modifiers. Give diagnostic criteria, if applicable	5-8
Data sources/ measurement	8*	For each variable of interest, give sources of data and details of methods of assessment (measurement). Describe comparability of assessment methods if there is more than one group	5-8
Bias	9	Describe any efforts to address potential sources of bias	18-19
Study size	10	Explain how the study size was arrived at	5
Quantitative variables	11	Explain how quantitative variables were handled in the analyses. If applicable, describe which groupings were chosen and why	5-8
Statistical methods	12	(*a*) Describe all statistical methods, including those used to control for confounding	8-9
		(*b*) Describe any methods used to examine subgroups and interactions	9
		(*c*) Explain how missing data were addressed	5
		(*d*) If applicable, explain how loss to follow-up was addressed	
		(*e*) Describe any sensitivity analyses	9
**Results**			
Participants	13*	(a) Report numbers of individuals at each stage of study—eg numbers potentially eligible, examined for eligibility, confirmed eligible, included in the study, completing follow-up, and analysed	4-5
		(b) Give reasons for non-participation at each stage	
		(c) Consider use of a flow diagram	
Descriptive data	14*	(a) Give characteristics of study participants (eg demographic, clinical, social) and information on exposures and potential confounders	9-11
		(b) Indicate number of participants with missing data for each variable of interest	Only included those with complete data
		(c) Summarise follow-up time (eg, average and total amount)	4-5

For peer review only - http://bmjopen.bmj.com/site/about/guidelines.xhtml

Outcome data	15*	Report numbers of outcome events or summary measures over time	9
Main results	16	(*a*) Give unadjusted estimates and, if applicable, confounder-adjusted estimates and their precision (eg, 95% confidence interval). Make clear which confounders were adjusted for and why they were included	11-14
		(*b*) Report category boundaries when continuous variables were categorized	11-12
		(*c*) If relevant, consider translating estimates of relative risk into absolute risk for a meaningful time period	
Other analyses	17	Report other analyses done—eg analyses of subgroups and interactions, and sensitivity analyses	11,14, supplements 4-7
**Discussion**			
Key results	18	Summarise key results with reference to study objectives	14-15
Limitations	19	Discuss limitations of the study, taking into account sources of potential bias or imprecision. Discuss both direction and magnitude of any potential bias	18-19
Interpretation	20	Give a cautious overall interpretation of results considering objectives, limitations, multiplicity of analyses, results from similar studies, and other relevant evidence	15-17, 19
Generalisability	21	Discuss the generalisability (external validity) of the study results	19
**Other information**			
Funding	22	Give the source of funding and the role of the funders for the present study and, if applicable, for the original study on which the present article is based	25

*Give information separately for exposed and unexposed groups.

Note: An Explanation and Elaboration article discusses each checklist item and gives methodological background and published examples of transparent reporting. The STROBE checklist is best used in conjunction with this article (freely available on the Web sites of PLoS Medicine at http://www.plosmedicine.org/, Annals of Internal Medicine at http://www.annals.org/, and Epidemiology at http://www.epidem.com/). Information on the STROBE Initiative is available at http://www.strobe-statement.org.

For peer review only
